# Long‐Term Psychodynamic Psychotherapy for Depression: A Systematic Review and Meta‐Analysis of Randomized Controlled Trials

**DOI:** 10.1155/da/6172409

**Published:** 2026-09-23

**Authors:** Max Moser, Angela Barrett, Christian. F. J. Woll-Weber, Peter Fonagy, Patrick Luyten, Chloe Campbell

**Affiliations:** ^1^ Research Department of Clinical, Educational and Health Psychology, University College London, London, UK, ucl.ac.uk; ^2^ Anna Freud, London, UK; ^3^ Clinical Child and Adolescent Psychology and Psychotherapy, Freie Universität Berlin, Berlin, Germany, fu-berlin.de; ^4^ Faculty of Psychology and Educational Sciences, KU Leuven, Leuven, Belgium, kuleuven.be

## Abstract

This pre‐registered systematic review and meta‐analysis (PROSPERO CRD42023490547) evaluated the effectiveness of long‐term psychodynamic psychotherapy (LTPP) in reducing depressive symptoms using the Grading of Recommendations Assessment, Development, and Evaluation (GRADE) framework and updated empirically supported treatment (EST) criteria. MEDLINE, PsycINFO, Embase, and the Cochrane Central Register of Controlled Trials were searched through January 16, 2025. Sixteen randomized controlled trials involving 1992 participants met inclusion criteria (LTPP ≥9 months, ≥30 sessions), encompassing varied populations, psychodynamic modalities, and comparator conditions. Effects were pooled using two‐ or three‐level random‐effects models. Risk of bias was assessed using Cochrane RoB 2. At treatment completion, LTPP showed significant reductions in depressive symptoms compared to all comparators (SMD = 0.34, 95% CI [0.20, 0.47]) and to active treatments specifically (SMD = 0.27, 95% CI [0.1, 0.45]). Effects were generally sustained at follow‐up. Subgroup analyses revealed larger effects when LTPP was compared to active controls (SMD = 0.61), but with greater heterogeneity and very low certainty of evidence. GRADE assessments indicated high‐certainty evidence for depressive symptom outcomes in the all‐trials condition and moderate‐certainty evidence in comparisons with active treatments. According to updated EST criteria, these findings support a Strong recommendation for LTPP in reducing depressive symptoms. However, limited evidence on functional outcomes, economic impact, and long‐term maintenance precluded a Very Strong recommendation. Evidence from comparisons with active controls was of very‐low certainty, supporting only a Weak recommendation. Risk of bias was a concern in most studies. Moderator analyses were prevented by small study numbers. Future individual patient data meta‐analyses are needed to identify predictors of treatment response and to establish which subgroups of patients with depression are most suitable candidates for LTPP. Funding: Royal Australian and New Zealand College of Psychiatrists.

## 1. Introduction

Major depressive disorder (MDD) remains a leading cause of disability globally, imposing substantial clinical and economic burdens [1]. While the effectiveness of first‐line pharmacotherapy and psychotherapy for mild to moderate depression is well established [2–4], most individuals with MDD exhibit insufficient response to these treatments. Approximately one‐third of patients do not achieve remission despite receiving multiple well‐implemented interventions. Notably, the nonresponse rate to brief psychotherapies is even higher, at around 40% to 60% [2, 3], and unlike pharmacological interventions, there are currently no validated protocols for sequencing psychotherapies [5]. Treatment nonresponse in depression is strongly linked to increased clinical severity, including recurrent depressive episodes [6–9], and each subsequent treatment failure escalates illness chronicity, functional impairment, and healthcare costs [10].

It is well established that mood disorder is not a unitary disorder [11]. Emerging clinical evidence highlights that a substantial subset of patients, variously labeled as “treatment resistant” [12], “difficult to treat” [13], or having “complex depression” [14], frequently exhibit comorbidity with other psychiatric disorders, notably anxiety [15, 16] and personality disorders (PDs) [17, 18].

Personality pathology is strongly associated with the onset and recurrence of depressive episodes, even at subthreshold levels, highlighting the close relationship between personality dysfunction and depression [19]. Longstanding epidemiological research has consistently supported a Strong association between mood disorder and personality problems [20, 21], particularly borderline PD (BPD) [22]. A meta‐analysis by Friborg et al. [23] estimated that approximately 50% of individuals with MDD fulfill diagnostic criteria for at least one PD, primarily from Clusters B and C. Conversely, cross‐sectional studies report depression prevalences ranging from 32% to 83% among individuals with BPD, with lifetime MDD prevalence estimated at 96% in this population [24]. The significant overlap between mood disorders and personality pathology is clinically highly relevant. A meta‐analysis from Newton‐Howes et al. [25] demonstrated that concurrent PDs more than doubled the likelihood of poor outcomes in depression treatment.

The high comorbidity rates among depressed patients, combined with limited advances in reliably delineating clinically meaningful depression subtypes [26], have encouraged the adoption of “complex depression” as a pragmatic umbrella term to describe depression complicated by factors that exacerbate severity or impede treatment responsiveness. Complex depression typically refers to depression occurring alongside personality dysfunction such as deficits in self‐organization, particularly affect regulation and socio‐cognitive functioning [14, 27]. In complex presentations, depressive symptoms are usually embedded within broader personality and interpersonal problems, likely necessitating specialized therapeutic approaches distinct from those employed for less complex depressive presentations [28, 29].

The limitations of both pharmacological interventions and brief psychotherapies underscore the need for further research into long‐term treatments. A longer treatment duration may be particularly important for individuals with complex psychopathology, including personality pathology, for whom brief interventions often yield limited benefits and may even be insufficient to establish the therapeutic conditions required for meaningful change [30]. Longer‐term psychotherapies, including extended variants of dialectical behavior therapy (DBT), schema‐focused therapy, and psychodynamic psychotherapy, have demonstrated efficacy for severe and complex mental disorders [31–34]. A plausible explanation for these effects is treatment duration itself, which allows sufficient time to establish a stable therapeutic alliance, work through entrenched cognitive‐affective and interpersonal patterns, and support more durable change in complex presentations [35]. These treatments may be particularly beneficial for patients with recurrent depression and comorbid PDs, populations known to experience heightened difficulties in forming therapeutic alliances [36, 37].

Long‐term psychodynamic psychotherapy (LTPP), a specific psychodynamic psychotherapy (PDT) variant, is one such approach supported by several randomized controlled trials (RCTs) for complex mental health conditions [38, 39]. PDT encompasses treatments targeting recurring intra‐ and interpersonal patterns, alongside a focus on characteristic defensive mechanisms employed by individuals with these conditions in relationships [40]. A central therapeutic component involves examining these dynamics as they arise within the therapist–client relationship, promoting insight and facilitating change.

In contrast to brief PDT, LTPP is typically defined by treatment durations exceeding 6 months and 24 sessions [41] or 12 months and 50 sessions [39, 42]. Nearly two decades ago, a seminal meta‐analysis by Leichsenring and Rabung [42] systematically assessed LTPP’s effectiveness, revealing moderate to large effect sizes across various symptomatic and psychosocial domains, including target problems, psychiatric symptoms, personality functioning, and social adjustment. These results have since been replicated in subsequent meta‐analyses [38, 39, 42, 43]. Nonetheless, no meta‐analysis has specifically investigated the relative effectiveness of LTPP for reducing depressive symptoms.

Advances in systematic evidence evaluation, notably the Grading of Recommendations Assessment, Development, and Evaluation (GRADE) framework [44, 45] and updated empirically supported treatment (EST) criteria by the American Psychological Association’s Society of Clinical Psychology [46], have raised standards for identifying ESTs in the meantime. These frameworks emphasize efficacy alongside evidence quality, clinical significance, and applicability in real‐world settings. Specifically, the GRADE framework categorizes evidence into high, moderate, low, or very low quality based on reviewers’ confidence in findings, while the revised EST criteria translate evidence into clinical recommendations grounded in sustained improvements and real‐world effectiveness [47]. A recent umbrella review [4] concluded that PDT warranted a Strong recommendation for depression treatment; however, the review did not clearly distinguish between brief PDT and LTPP studies. Thus, to date, no comprehensive meta‐analysis explicitly evaluating LTPP’s efficacy in reducing depression symptoms according to these contemporary standards has been conducted.

The current study presents a pre‐registered systematic review and meta‐analysis (CRD42023490547; data and analysis code openly accessible at https://osf.io/85p4q/) of RCTs evaluating LTPP in adults with clinically significant depressive symptoms, including, but not limited to, presentations characterized by clinical and/or structural complexity, such as depression co‐occurring with BPD, other PDs, anxiety disorders, or treatment‐resistant depression.

As noted, patients presenting with depression often display a range of clinical features that cut across traditional diagnostic boundaries. The aim of the present paper is, therefore, not to collapse diagnostic categories, but to also draw attention to individuals whose depressive symptoms may also co‐occur with anxiety, personality pathology, suicidality, self‐harm, and trauma histories. Patients with more complex and severe presentations are frequently, but not always, excluded from psychotherapy and pharmacotherapy trials because these factors complicate brief interventions. However, such characteristics are also associated with the high nonresponse rates commonly observed in depression trials [2, 3], likely partly reflecting the impact of comorbid personality pathology on treatment outcomes.

Individuals with these complicating features are often only later appropriately diagnosed with PDs in which depression is a prominent feature, highlighting the substantial overlap between depressive and personality pathology. Conceptualizing depression dimensionally, as ranging from transient episodes to more ingrained, personality‐linked forms, is also consistent with contemporary dimensional models of psychopathology, such as the Hierarchical Taxonomy of Psychopathology [48]. Within a dimensional framework, depressive symptoms occurring in the context of personality pathology can be understood as part of a broader phenotype, sometimes called “complex depression,” that is characterized by greater chronicity, comorbidity, and interpersonal impairment. This perspective supports the inclusion of clinically complex populations in research on depression, as such presentations are common in routine clinical practice. Consequently, studying these populations may enhance the ecological validity and generalizability of findings, while also underscoring the potential need for more individualized and longer‐term treatment approaches.

The research aims of this review were organized hierarchically. The primary research aim was to estimate the effect of LTPP on depressive symptom severity at treatment completion, including subgroup analyses comparing LTPP with active treatments and active control conditions. Secondary aims were to examine the durability of depressive symptom change at follow‐up and to explore effects on secondary clinical and psychosocial outcomes. Within‐group analyses were also conducted and treated as descriptive and hypothesis‐generating. Lastly, we evaluated the certainty and clinical recommendation status of the evidence for LTPP in reducing depressive symptoms using the GRADE framework and updated EST criteria, which is now considered an essential component of a broader state‐of‐the‐art assessment of the effectiveness of psychological treatments. Employing contemporary methodological evaluative frameworks, this study provides a comprehensive synthesis of the empirical evidence for LTPP in the reduction of depressive symptoms and clarifies its status as an EST under the current guidelines.

## 2. Methods

### 2.1. Selection Criteria

This review follows PRISMA guidelines, with a completed 2020 PRISMA Checklist available in the Supporting Information, as shown in Table S1. Our selection criteria were specified according to the following population, intervention, comparator, outcome, and study design (PICOS) framework. Participants were adults experiencing depression, including those with “complex” presentations. Complex depression was defined as meeting clinical thresholds on validated depression measures and exhibiting features that complicate treatment response, such as comorbid personality, substance use, or anxiety disorders. Trials of PDs where comorbid depression was not explicitly stated were included only when depression symptoms were continuously measured and mean depression scores for both treatment arms met or exceeded clinical cut‐off values, such as scores of ≥17 on the Hamilton Depression Rating Scale (HDRS) [49] or scores of ≥14 on the Beck Depression Inventory‐II (BDI‐II) [50].

We use the term “complex depression” pragmatically rather than nosologically to refer to clinically significant depressive symptoms occurring in the context of additional factors that complicate treatment and prognosis, including chronicity, treatment resistance, personality pathology, interpersonal dysfunction, trauma‐related symptoms, substance misuse or other psychiatric comorbidity. The review does not seek to validate complex depression as a discrete diagnostic category; rather, it synthesizes evidence relevant to the kinds of complex depressive presentations for which LTPP is commonly considered in clinical practice.

Interventions were LTPP, referring to the long‐term variant of PDT. PDT refers to a number of treatment approaches undergirded by a common theoretical orientation, each having in common a focus on emotion and the exploration of emotional avoidance and defensive patterns, the investigation and identification of patterns of relating to the self and others (including the therapeutic relationship), and a focus on how past experiences contribute to present functioning [51–53]. PDT integrates supportive, mentalizing and interpretive techniques. The balance between these depends on the individual’s psychological needs and capacities. Interpretive interventions aim to deepen understanding of recurrent internal conflicts underlying distress, while supportive techniques focus on reinforcing coping mechanisms and psychosocial functioning, particularly when these capacities are compromised. Mentalizing interventions aim to foster the patient’s capacity to reflect on the self and others. LTPP was defined as PDTs lasting ≥ 9 months and comprising ≥ 30 sessions. Given the variability in definitions of LTPP in the literature, our cutoff was chosen as a midpoint between the two most authoritative definitions: Gabbard’s threshold of 6 months or 24 sessions [41] and Leichsenring and Rabung’s criterion of 12 months or 50 sessions [39, 42].

Comparators included both active controls and active treatments. Active controls were defined as nonmanualized treatment as usual (TAU) in any setting, pharmacotherapy, or mixed community‐based interventions (e.g., unstructured or mixed‐modality psychotherapy). Active treatments were defined as manualized, evidence‐based psychotherapeutic interventions or structured models of care (e.g., cognitive behavioral therapy [CBT], DBT, short‐term PDT, and structured clinical management [SCM]). Outcomes included depressive symptom severity (primary outcome) and secondary outcomes such as anxiety, global distress, interpersonal functioning, quality of life, and clinical events. Study design was limited to RCTs. No restrictions were placed on language or publication date.

### 2.2. Search Strategy

Our search spanned the following databases: MEDLINE (from January 1, 1946, to January 16, 2025), PsycINFO (from January 1, 1806, to January 16, 2025), Embase (from January 1, 1974, to January 16, 2025), and the Cochrane Controlled Register of Trials (from its inception until January 16, 2025). Our search terms, which are supplied on OSF (https://osf.io/unpgf), incorporated an established search filter for RCTs that has undergone rigorous testing by the team at the Canadian Agency for Drugs and Technologies in Health Research Information Services [54]. Moreover, our search results were cross‐referenced with a regularly updated and freely accessible comprehensive list of RCTs for PDT [55]. Additionally, we engaged PDT experts to identify unpublished or inaccessible data. Efforts were made to include gray literature and non‐English studies, with translations sought where necessary, to minimize publication bias and enhance inclusivity.

### 2.3. Data Extraction and Risk of Bias Assessment

**Figure 1:**
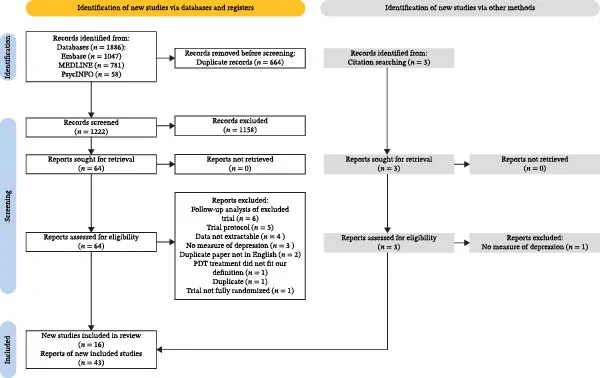
PRISMA flow diagram of study screening and selection. *Note:* PRISMA flow diagram created using the Shiny app developed by Haddaway et al. [56].

Titles and abstracts from the systematic search were independently screened by two reviewers (Max Moser and Angela Barrett), with discrepancies resolved via consensus or by consulting a third reviewer (Chloe Campbell) following the full‐text review. Data extraction was independently completed by Max Moser and Angela Barrett using structured Excel forms capturing the first author, publication year, interventions, target diagnoses, participant numbers, outcome measures, and outcome scores (means and standard deviations). Data consistency across forms was cross‐checked, and any discrepancies were resolved through joint discussion. A PRISMA flow diagram of the study screening and selection process is presented in Figure 1.

**Figure 2:**
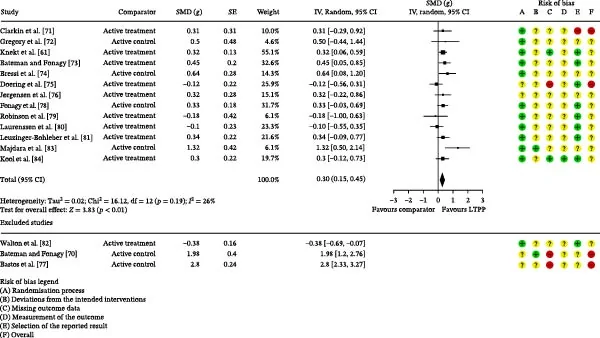
Forest plot of between‐group effects of long‐term psychodynamic psychotherapy on depressive symptoms at end of treatment (outliers excluded), with risk of bias summary.

The risk of bias was assessed using the Cochrane RoB2 tool [57], evaluating RCTs on randomization processes, intervention deviations, missing outcome data, outcome measurement, and selective reporting (Figure 2).

### 2.4. Outcomes

Primary outcomes focused on depression severity, with secondary outcomes including anxiety severity and global distress, measured via observer‐rated (e.g., HDRS) and self‐report (e.g., BDI) instruments. Additional secondary measures encompassed interpersonal functioning, clinical events (hospitalizations and emergency visits), and quality of life assessed using observer‐rated and self‐report tools. Means and standard deviations were extracted at treatment completion and at all available follow‐up points. Summary standardized mean differences (SMDs) were computed for within‐ and between‐group effects at treatment completion and at follow‐up intervals of 3–6, 12–18, and 24–36 months. An aggregate summary across all timepoints was also calculated. Data beyond a 5‐year follow‐up were excluded to minimize effects of external factors unrelated to treatment.

### 2.5. Statistical Analysis

Meta‐analytic models estimated absolute within‐group (primary outcomes) and relative between‐group (all outcomes) effects. Subgroup analyses compared LTPP to active controls (nonmanualized TAU, pharmacotherapy, or community‐based interventions such as unstructured psychotherapy) and active treatments (manualized evidence‐based psychotherapies or structured care models). To assess the robustness of pooled estimates, we conducted analyses to identify and exclude statistical outliers. Outliers were defined as individual study effects whose 95% confidence intervals (CIs) did not overlap with the pooled summary estimate for a given model. When identified, these studies were excluded from the primary meta‐analytic models reported in tables, while full models including all studies were retained and reported in Section 3. Outliers identified in models of the pooled effect on depression severity (the critical outcome) were also excluded when estimating pooled effects for secondary outcomes.

Meta‐analyses employed two‐level random‐effects models and, where possible, three‐level correlated and hierarchical effects (CHE) models using the “metafor” package in R version 4.3.1 [58, 59]. SMDs (Hedges’s *g*) with 95% CIs were calculated for each outcome. Where outcomes were assessed by multiple instruments (e.g., BDI and HDRS), all relevant data were integrated using three‐level models. Between‐group SMDs at treatment completion are illustrated in forest plots, while all other summary estimates and subgroup analyses are presented in tables. SMD values were reverse‐scored such that positive values indicate effects favoring LTPP.

Although not pre‐registered, to aid interpretation of effect sizes, we also report numbers‐needed‐to‐treat (NNT) estimates for all between‐group comparisons. Owing to the absence of consistently reported binary outcomes (e.g., response or remission rates), we employed the Kraemer and Kupfer [60] method, which estimates NNT by first converting the SMD into the area under the receiver operating curve and then translating this into an NNT value. This approach yields the number of patients who would need to receive LTPP, rather than the comparator, for one additional patient to benefit. Notably, this benefit reflects a probabilistic shift in the outcome distribution favoring LTPP, rather than a discrete clinical improvement defined by categorical thresholds such as response or remission.

To address heterogeneity arising from varied control conditions, subgroup analyses separately compared LTPP versus active controls and active treatments. In addition, a sensitivity analysis was conducted including only trials in which all participants were diagnosed with depression using structured diagnostic interviews (e.g., the Structured Clinical Interview for DSM Disorders (SCID)). While all included trials required baseline depression scores above validated clinical thresholds, this analysis assessed the robustness of treatment effects when the included trials were restricted to a diagnostically homogeneous subset. The trial from Knekt et al. [61], where 85% of participants received a SCID diagnosis of depression, was deemed sufficiently diagnostically homogenous and was retained in this sensitivity analysis.

Prediction intervals were calculated to estimate the expected range of effect sizes for future studies [62]. Heterogeneity was quantified using the *I* ^2^ statistic, and in CHE models, both within‐study and between‐study heterogeneity components (*τ* ^2^ and *I* ^2^) were reported. When means and standard deviations were unavailable, effect sizes were derived using standard conversion methods detailed in the Supporting Information (Supporting Information, Section 1). Effect sizes were categorized according to Cohen’s [63] criteria: negligible (SMD = 0.00–0.19), small (SMD = 0.20–0.49), moderate (SMD = 0.50–0.79), large (SMD = 0.80–1.29), and very large (SMD ≥1.29). An SMD ≥0.24 indicated a minimal important difference (MID) [64], with SMD ≥0.50 representing clinically meaningful effects.

Hierarchical models accounted for nested effect sizes within studies, enabling the inclusion of multiple instruments and multiarm trials, thereby preserving statistical power and minimizing biases arising from non‐independence [65]. Within‐study dependencies were modeled using a covariance matrix with a fixed correlation coefficient (*ρ* = 0.80), following recommendations by Tanner‐Smith and Tipton [66]. Robust variance estimation (RVE) with CR‐2 corrections and Satterthwaite degrees of freedom adjustments was employed using the “clubSandwich” package in R to yield accurate standard errors, even in cases of model misspecification [67]. When Satterthwaite degrees of freedom dropped below 4, estimates were considered unstable, and thus, two‐level models were applied. In these instances, within‐study effect sizes were aggregated using a compound symmetric variance‐covariance matrix, imputing the fixed correlation coefficient (*ρ* = 0.80) within the “metafor” package in R [59].

Publication bias was examined using the Egger Sandwich and three‐parameter selection model (3PSM) likelihood ratio tests, methods particularly suitable for multilevel meta‐analytic models [68].

### 2.6. Quality Assessment

Study quality was assessed using the GRADE system [44, 45] and the updated EST criteria from the American Psychological Association’s Society of Clinical Psychology [46, 69]. GRADE categorizes evidence quality as high, moderate, low, or very low based on methodological limitations, consistency of findings, and CI precision, with high‐quality evidence indicating Strong confidence that the observed effect closely matches the true effect. The updated EST criteria further translate evidence into clinical recommendations classified as Very Strong, Strong, or Weak, integrating the quality of evidence with clinical significance (symptom improvement and functional outcomes), durability of effects, and real‐world applicability. A Very Strong recommendation requires high‐quality evidence demonstrating clinically meaningful effects on both symptoms and functioning, sustained for at least 3 months post‐treatment, supported by at least one effectiveness study in a non‐research setting. A Strong recommendation indicates moderate‐ to high‐quality evidence for clinically meaningful improvement in either symptoms or functioning, whereas a Weak recommendation applies when evidence of clinical benefit is derived from low‐ or very‐low‐quality studies. EST recommendations were assigned only for depressive symptom severity, the prespecified critical outcome. Secondary clinical and functional outcomes informed the overall evaluation but were not assigned separate EST recommendations.

## 3. Results

### 3.1. Search Results

#### 3.1.1. Study Selection

The systematic search yielded 1222 nonduplicate reports, which were independently screened by two reviewers. Of these, 64 underwent full‐text assessment, resulting in the inclusion of 43 reports from 16 RCTs (refer to the PRISMA flow diagram, Figure 1). The characteristics of included studies are tabulated and reported in Table 1.

**Table 1:** Characteristics of included randomized controlled trials.

Author, year (country)	Target disorder	LTPP arms	Setting	Treatment format	Session frequency	Comparators	Duration (months)	Follow‐up available	Risk of bias	Subgroup
Bateman and Fonagy, 1999 [70] (UK)	BPD	MBT (*n* = 19)	Day hospital programme	Group + individual	Multiple weekly sessions	TAU (*n* = 19)	18	Yes	High	Active ctl
Clarkin et al., 2007 [71] (US)	BPD	TFP (*n* = 31) SPT (*n* = 30)	Outpatient specialist PD clinic	Individual	2 weekly	DBT (*n* = 29)	12	No	High	Active tx
Gregory et al., 2008 [72] (US)	BPD	DDP (*n* = 15)	Outpatient clinic	Individual	1 weekly	TAU (*n* = 15)	12	Yes	Concerns	Active ctl
Knekt et al., 2008 [61] (Finland)	Depression and anxiety	LTPP (*n* = 128)	Outpatient psychotherapy clinics	Individual	2–3 weekly	STPP (*n* = 101), SFT (*n* = 97)	36	Yes	Concerns	Active tx
Bateman and Fonagy, 2009 [73] (UK)	BPD	MBT (*n* = 71)	Specialist outpatient MBT programme	Group + individual	1 individual + 1 group weekly	SCM (*n* = 63)	18	Yes	Concerns	Active tx
Bressi et al., 2010 [74] (Italy)	Depression and anxiety	Brief dynamic (*n* = 30)	Outpatient psychiatric clinic	Individual	1 weekly	TAU (*n* = 30)	12	No	Concerns	Active ctl
Doering et al., 2010 [75] (GER/AT)	BPD	TFP (*n* = 52)	Outpatient psychotherapy practices	Individual	2 weekly	Community psychotherapy (*n* = 52)	12	No	High	Active ctl
Jørgensen et al., 2013 [76] (DK)	BPD	MBT (*n* = 74)	Outpatient MBT programme	Group + individual	1 individual + 1 group weekly	Supportive group (*n* = 37)	24	Yes	Concerns	Active tx
Bastos et al., 2015 [77] (Brazil)	Depression	LTPP (*n* = 90), LTPP + fluoxetine (*n* = 91)	Outpatient psychiatric clinic	Individual	1 weekly	Fluoxetine (*n* = 90)	24	No	High	Active ctl
Fonagy et al., 2015 [78] (UK)	TRD	LTPP + TAU (*n* = 67)	Specialist outpatient psychotherapy service	Individual	1 weekly	TAU (*n* = 62)	18	Yes	Concerns	Active ctl
Robinson et al., 2016 [79] (UK)	ED/BPD	MBT‐ED (*n* = 34)	Multisite NHS eating disorder/personality disorder services	Group + individual	1 individual + 1 group weekly	Supportive ED (*n* = 34)	12	Yes	Concerns	Active tx
Laurenssen et al., 2018 [80] (NL)	BPD	MBT‐DH (*n* = 54)	Day hospital MBT programme	Group + individual	Multiple sessions weekly	S‐TAU (*n* = 41)	18	Yes	Concerns	Active tx
Leuzinger‐Bohleber et al., 2019 [81] (GER)	Chronic depression	Psychoanalysis (*n* = 47)	Specialist psychoanalytic outpatient clinics	Individual	NR; mean 80.4 sessions in year 1 and 234 over 3 years	Long‐term CBT (*n* = 47)	36	No	Concerns	Active tx
Walton et al., 2020 [82] (AUS)	BPD + SI/NSI	Conversational Model (*n* = 83)	Public outpatient mental health service	Individual	2 weekly	DBT (*n* = 83)	14	No	Concerns	Active tx
Majdara et al., 2021 [83] (Iran)	BPD	DDP (*n* = 15)	University outpatient psychology clinic	Individual	1 weekly	Enhanced TAU (*n* = 15)	12	No	Concerns	Active ctl
Kool et al., 2024 [84] (NL)	Depression + PD	STPSP‐50 (*n* = 54)	Specialist personality disorder treatment center	Individual	2 initially, then 1 weekly^a^	STPSP‐25 (*n* = 68), ST‐50 (*n* = 60), ST‐25 (*n* = 64)	12	No	Concerns	Active tx

*Note*: Community, experienced community therapists; High, high risk of bias (both according to RoB2 classification).

Abbreviations: AT, Austria; AUS, Australia; BPD, borderline personality disorder; BR, Brazil; CBT, cognitive behavioral therapy; Concerns, some concerns about risk of bias; ctl, control; DBT, dialectical behavior therapy; DDP, dynamic deconstructive psychotherapy; DK, Denmark; ED, eating disorder; Enhanced TAU, enhanced treatment as usual; FIN, Finland; Fluox, fluoxetine; FU, follow‐up; GER, Germany; IR, Iran; IT, Italy; LTPP, long‐term psychodynamic psychotherapy; MBT, mentalization‐based treatment; MBT‐DH, mentalization‐based treatment (day hospital); MBT‐ED, mentalization‐based treatment for eating disorders; mo, months; NL, Netherlands; NR, not reported; NSI, nonsuicidal self‐injury; PD, personality disorder; SCM, structured clinical management; SFT, solution‐focused therapy; SI, suicidal ideation; Spec‐TAU, specialist treatment as usual; SPT, supportive treatment; ST, schema therapy; STPP, short‐term psychodynamic psychotherapy; STPSP, short‐term psychodynamic supportive psychotherapy; TAU, treatment as usual; TFP, transference‐focused psychotherapy; TRD, treatment‐resistant depression; tx, treatment; UK, United Kingdom; US, United States.

^a^Session frequency was twice weekly during the initial phase of treatment and reduced to once weekly during the later phase according to the study protocol.

#### 3.1.2. Characteristics of Included Studies

The 16 included RCTs encompassed 18 LTPP arms evaluating a range of psychodynamic approaches. The most frequently evaluated modality was mentalization‐based treatment (MBT; *k* = 5: [70, 73, 76, 79, 80]). Three trials evaluated LTPP [61, 77, 78], with Bastos et al. [77] contributing two LTPP arms (LTPP alone and LTPP combined with fluoxetine). Transference‐focused psychotherapy (TFP; *k* = 2: [71, 75]) and dynamic deconstructive psychotherapy (DDP; *k* = 2: [72, 83]) were each represented by two trials. One trial each evaluated traditional psychoanalytic treatment [81], supportive psychodynamic treatment [71], and the conversational model [82]. Two additional trials met inclusion criteria despite being described by their authors as brief or short‐term psychodynamic treatments [74, 84]. The impact of including these trials on the pooled effect for the primary outcome was assessed through sensitivity analyses.

Across trials, nine compared LTPP against an active treatment (e.g., an alternative manualized psychotherapy), and seven compared LTPP against an active control condition (e.g., TAU). Control conditions most commonly involved TAU (*k* = 7), including enhanced and specialist TAU variants ([70, 72, 74, 77, 78, 80, 83]). Other comparators included DBT (*k* = 2: [71, 82]), SCM (*k* = 1: [73]), community psychotherapy (*k* = 1: [75]), schema therapy (*k* = 1: [84]), CBT (*k* = 1: [81]), fluoxetine (*k* = 1: [77]), supportive group therapy (*k* = 1: [76]), short‐term psychodynamic psychotherapy (STPP; *k* = 1: [61]), and solution‐focused therapy (SFT; *k* = 1: [61]). Most studies employed a single comparator condition (*k* = 12), while four trials included multiple comparator or psychodynamic arms [61, 71, 77, 84].

#### 3.1.3. Clinical Populations and Intervention Delivery

The included trials enrolled adults with clinically significant depressive symptoms across a spectrum of diagnostic complexity. Ten studies recruited patients with PDs—predominantly BPD—in whom depression was prominent at the group level [70–73, 75, 76, 79, 80, 82, 83], with Kool et al. [84] explicitly recruiting patients with comorbid depressive disorder and PD. Two studies focused on chronic or treatment‐resistant depression [78, 81], while the remaining trials enrolled primary depressive or mixed anxiety‐depressive presentations [61, 74, 77].

The treatment duration ranged from 12 to 36 months. Seven trials were conducted over 12 months [71, 72, 74, 75, 79, 83, 84], one over 14 months [82], four over 18 months [70, 73, 78, 80], two over 24 months [76, 77], and two over 36 months [61, 81]. The settings, treatment format, and intensity varied considerably. Eleven trials were delivered as individual outpatient psychotherapy, with session frequency ranging from once weekly [72, 74, 77, 78, 83] to twice weekly [71, 75, 82] or two to three times weekly [61], one trial with an initially twice‐weekly frequency reducing to once weekly [84], and one high‐intensity trial in which session frequency was not formally reported but averaged 80.4 sessions in year one and 234 sessions across the full 3‐year treatment period [81]. Three trials were delivered in specialist outpatient MBT programs combining once‐weekly individual and once‐weekly group sessions [73, 76, 79]. Two trials were conducted within intensive day‐hospital programs delivering combined individual and group psychotherapy across multiple sessions weekly [70, 80]. Trials were conducted predominantly within European healthcare systems, although trials from the United States [71, 72], Australia [82], Iran [83], and Brazil [77] were also included.

### 3.2. Risk of Bias and Treatment Fidelity

The risk of bias, assessed via the RoB2 tool, indicated that for the randomization process, *K* = 11 trials (69%) were judged to be at low risk, while *K* = 5 (31%) had some concerns. Regarding deviations from intended interventions, *K* = 3 trials (19%) had low risk, whereas *K* = 13 (81%) had some concerns. For missing outcome data, *K* = 1 trial (6%) had low risk, *K* = 12 (75%) had some concerns, and *K* = 3 (19%) were judged to be at high risk. With respect to outcome measurement, *K* = 1 trial (6%) was rated as low risk and *K* = 15 (94%) as having some concerns. In terms of selective reporting, *K* = 10 trials (63%) showed low risk, *K* = 5 (31%) had some concerns, and *K* = 1 (6%) was rated as high risk. Overall, *K* = 12 trials (75%) were judged to have some concerns regarding the risk of bias, and *K* = 4 (25%) were rated as high risk. Treatment fidelity was separately evaluated in accordance with the revised EST criteria [47]: manualized treatments were used in *K* = 13 trials (81%), experienced therapists delivered treatment in *K* = 14 trials (88%), supervision‐based monitoring was reported in *K* = 12 trials (75%), and empirical assessment of treatment integrity was conducted in *K* = 10 trials (63%).

### 3.3. Medication Use and Control

In nearly all included trials, most participants commenced treatment on psychotropic medication. Only one trial excluded medication entirely or randomized to medication‐only versus no‐medication arms [77]. Full control of pharmacotherapy was implemented in two trials: one randomized participants to psychotherapy only, medication (fluoxetine) only, or combined therapy [77], and a second mandated selective serotonin reuptake inhibitor or serotonin–norepinephrine reuptake inhibitor initiation for all participants [74], irrespective of allocation. Four additional studies implemented partial controls [70, 73, 78, 81], including standardized dosing protocols or statistical adjustment for baseline use [81]. Ten of the 16 trials (63%) reported baseline antidepressant use separately for experimental and control arms; in each of these, the absolute difference between groups was ≤10 percentage points. Nine reported longitudinal medication trajectories, but only five formally tested between‐arm differences in these trajectories. Of those, the only statistically significant reductions in medication continuation occurred in two MBT trials—38% vs. 78% at 18 months (*p*  < 0.03; [70]) and 29.6% vs. 57.1% at 18 months (*p*  < 0.05; [73]). These findings suggest that the favorable outcomes of LTPP are not solely attributable to concurrent pharmacotherapy. Where directly tested, LTPP showed superiority over medication‐only comparators (SMD > 0.5; refer to Figure 2 [74, 77]. Due to inconsistent reporting and the absence of patient‐level data, we could not include medication use as a moderator in our meta‐analytic models.

### 3.4. Primary Outcome: Depressive Symptom Severity at Treatment Completion

#### 3.4.1. Between‐Group Effects Across All Trials

Given the substantial variability in the definition and intensity of comparator conditions—particularly TAU, which ranged from minimal care to structured or mixed manualized interventions—we first estimated a pooled effect across all trials to obtain a global estimate, followed by pre‐specified subgroup analyses. Across all eligible trials, LTPP demonstrated a statistically significant reduction in depressive symptom severity relative to treatment completion (*K* = 16, SMD = 0.57, 95% CI [0.08, 1.05]). Heterogeneity was substantial (*I* ^2^ = 94.85%), predominantly attributable to the between‐study variance (88%). Outlier diagnostics identified three trials [70, 77, 82] whose effects did not overlap with the pooled estimate and which were subsequently excluded.

Following their exclusion, the pooled effect was reduced but remained statistically and clinically meaningful (*K* = 13, SMD = 0.30, 95% CI [0.15, 0.44]), with markedly lower heterogeneity (*I* ^2^ = 21%), now entirely attributable to within‐study variance and reflecting increased consistency across trials. The prediction interval (0.02–0.58) indicated that future trials would be expected to observe negligible‐to‐moderate effects favoring LTPP. Excluding studies rated at a high risk of bias yielded an essentially unchanged estimate (*K* = 11, SMD = 0.34, 95% CI [0.20, 0.47]), with similar precision. Figure 2 presents individual study effects alongside a pooled effect estimate (excluding outliers) for depressive symptom reduction at treatment termination, accompanied by a summary of the risk of bias assessments (Table 1).

#### 3.4.2. Sensitivity Analyses

Two further sensitivity analyses were conducted. The first, which excluded trials originally described as “short‐term” [84] or “brief” PDT [74] by their authors, exhibited a comparable effect size (*K* = 9, SMD = 0.31, 95% CI [0.15, 0.48], *p* = 0.005) with widened CIs. Model fit favored the sensitivity model (AIC: full model = 6.40; sensitivity model = 1.67; ΔAIC = 4.73), suggesting better parsimony [85]. The second analysis was restricted to trials in which all participants were diagnosed with depression using structured diagnostic interviews (e.g., SCID). This subset showed a comparable effect size to the full model (*K* = 5, SMD = 0.36, 95% CI [0.18, 0.54], *p*  < 0.001).

#### 3.4.3. Subgroup Comparisons

Subgroup analyses separately assessed the effects of LTPP against active controls (*K* = 7) and active treatments (*K* = 9). Table 2 summarizes these subgroup findings for depression severity, excluding statistical outliers. Limited data within subgroups prevented follow‐up interval‐specific analyses, and small subgroup sizes precluded modeling secondary outcomes.

**Table 2:** Between‐group effects of LTPP on severity of depression excluding outliers.

Timepoint	*K* (no. effects)	Satt df^a^	SMD^b^	NNT	95% CI	95% PI	*p*	Heterogeneity				
								Within‐study		Between‐study		Overall
								*τ* ^2^	*I* ^2^ (%)	*τ* ^2^	*I* ^2^ (%)	*I* ^2^ (%)
Across all trials^c^												
End of treatment	13 (21)	7.85	0.30	6	0.15, 0.44	0.02, 0.58	0.001 ^∗∗^	0.011	21.08	0.00	0.00	21.08
End of treatment—high RoB studies removed^d^	11 (18)	6.34	0.34	6	0.20, 0.47	0.06, 0.61	0.001 ^∗∗^	0.010	20.76	0.00	0.00	20.76
Follow‐up (3–6 months)	4 (5)	—	0.43	5	0.00, 0.85	0.00, 0.85	0.05 ^∗^	—	—	—	—	0.00
Follow‐up (12–18 months)	5 (8)	—	0.28	7	0.06, 0.51	0.06, 0.51	0.03 ^∗^	—	—	—	—	0.00
Follow‐up (24–36 months)	3 (8)	—	0.37	5	−0.31, 1.06	−0.81, 1.55	0.14	—	—	—	—	66.73
All time‐points	13 (46)	7.27	0.28	7	0.14, 0.42	−0.03, 0.59	0.002 ^∗∗^	0.014	29.62	0.00	0.00	29.62
All time‐points—high RoB studies removed^d^	11 (43)	5.92	0.31	6	0.17, 0.46	−0.01, 0.64	0.002 ^∗∗^	0.014	30.24	0.00	0.00	30.24
LTPP versus active controls^c^												
End of treatment	6 (7)	4.91	0.72	3	−0.06, 1.50	−1.19, 2.63	0.065	0.012	2.31	0.44	85.49	87.81
End of treatment—high RoB studies removed^d^	4 (5)	—	0.61	4	−0.03, 1.25	−0.45, 1.66	0.06	—	—	—	—	45.16
All time‐points	6 (17)	4.74	0.62	3	0.05, 1.19	−0.72, 1.96	0.04 ^∗^	0.039	14.74	0.18	65.71	80.45
All time‐points—high RoB studies removed^d^	4 (13)	—	0.58	4	0.08, 1.09	−0.14, 1.30	0.04 ^∗^	—	—	—	—	24.31
LTPP versus active treatments^c^												
End of treatment	8 (14)	4.74	0.28	7	0.13, 0.43	0, 0.56	0.006 ^∗∗^	0.008	18.86	0.00	0.00	18.86
End of treatment—high RoB studies removed^d^	7 (13)	4.14	0.27	7	0.1, 0.45	−0.03, 0.58	0.01 ^∗^	0.009	20.56	0.00	0.00	20.56
All time‐points	8 (31)	4.44	0.23	8	0.1, 0.37	−0.07, 0.53	0.008 ^∗∗^	0.010	25.32	0.00	0.00	25.32
All time‐points—high RoB studies removed^d^	7 (30)	—	0.24	8	0.07, 0.4	0.07, 0.4	0.01 ^∗^	—	—	—	—	0.00

*Note: K*, number of clusters (i.e., total number of effects within clusters); Satt df, Satterthwaite degrees of freedom; SMD, standardized mean difference, expressed as Hedges’s *g*; *τ* ^2^, variance component, where *τ* ^2^ (within‐study) represents the variance of true effects within clusters, and *τ* ^2^ (between‐study) represents between‐study heterogeneity; *I* ^2^, proportion of variance due to heterogeneity rather than sampling error, where *I* ^2^ (within‐study) represents within‐cluster heterogeneity, and *I* ^2^ (between‐study) represents between‐study heterogeneity.

Abbreviations: 95% CI, 95% confidence interval; 95% PI, 95% prediction interval; NNT, number needed to treat; RoB, risk of bias.

^a^Satt df value indicates a three‐level model in which variance is partitioned at the cluster level.

^b^Effect sizes have been reverse‐scored so that positive values favor LTPP.

^c^Bateman and Fonagy [70], Bastos et al. [77], and Walton et al. [82] were excluded as outliers from all‐trials models; Bastos et al. [77] was excluded from active control models; and Walton et al. [82] from active treatment models.

^d^Doering et al. [75] and Clarkin et al. [71] were excluded from all‐trials models due to high risk of bias; Bateman and Fonagy [70] and Doering et al. [75] from active control models; and Clarkin et al. [71] from active treatment models.

^∗^ *p*  < 0.05.

^∗∗^ *p*  < 0.01.

^∗∗∗^ *p*  < 0.001.

##### 3.4.3.1. LTPP Versus Active Controls

At treatment completion, LTPP demonstrated a large but imprecise effect relative to active controls (*K* = 7, SMD = 1.15, 95% CI [–0.01, 2.32]), accompanied by very high heterogeneity (*I* ^2^ = 96.0%), largely attributable to between‐study variance (*I^2^* between = 79.8%). Exclusion of an outlying study [77] reduced the pooled effect to a moderate magnitude (*K* = 6, SMD = 0.72, 95% CI [–0.06, 1.50]), although heterogeneity remained substantial (*I^2^* = 87.8%). Prediction intervals were wide, indicating considerable uncertainty and cross‐study variability. Excluding high‐risk‐of‐bias studies further attenuated this effect, yielding a moderate and nonsignificant estimate, reflecting sensitivity to study quality.

##### 3.4.3.2. LTPP Versus Active Treatments

In contrast to the active control subgroup, comparisons with active treatments showed small effects at treatment completion. The initial pooled estimate was negligible and nonsignificant (*K* = 9, SMD = 0.17, 95% CI [–0.08, 0.42], *p* = 0.15). Removal of an outlier [82] yielded a small but statistically significant effect (*K* = 8, SMD = 0.28, 95% CI [0.13, 0.43], *p* = 0.006), with low residual heterogeneity (*I* ^2^ = 18.86%) and no between‐study variance. Wide prediction intervals around this effect (0–0.56) demonstrate wide variability in expected effects in future trials. The pooled effect was essentially unchanged after excluding high‐risk studies.

### 3.5. Follow‐Up Effects on Depressive Symptom Severity

Table 2 summarizes between‐group effects for depressive symptom severity at treatment completion and across follow‐up intervals, following outlier removal. Owing to limited Satterthwaite degrees of freedom, multilevel models were restricted to end‐of‐treatment and aggregated analyses; two‐level models were applied for interval‐specific follow‐up estimates. Fewer than half of studies reported follow‐up data (*K* = 7, 43.8%), warranting cautious interpretation of durability estimates.

#### 3.5.1. Across All Trials

When pooled across all trials and comparator conditions, LTPP demonstrated small but statistically significant reductions in depressive symptoms across all timepoints (*K* = 13, SMD = 0.28, 95% CI [0.14, 0.42]). Heterogeneity was low to moderate (*I* ^2^ = 29.6%) and primarily attributable to within‐study variance. Interval‐specific analyses indicated sustained benefits at 3–6 and 12–18 months, whereas longer‐term estimates were positive but less precise.

#### 3.5.2. Subgroup Effects

When pooled across all timepoints, effects favored LTPP in both subgroup comparisons. Relative to active controls, effects were moderate with wide CIs and substantial heterogeneity (*K* = 6, SMD = 0.62, 95% CI [0.05, 1.19], *I* ^2^ = 80%). In contrast, comparisons with active treatments showed small but statistically significant and more consistent effects (*K* = 8, SMD = 0.23, 95% CI [0.10, 0.37], *I* ^2^ = 25%). Findings were similar after excluding high‐risk‐of‐bias studies.

### 3.6. Between‐Group Effects on Secondary Clinical Outcome and Psychosocial Functioning

#### 3.6.1. Secondary Clinical Outcome

Anxiety and global distress were examined as secondary clinical outcomes. As with the primary outcome, pooled effects were first estimated across all trials to derive global estimates, followed by comparator‐specific subgroup analyses. Clinical events were also examined; however, the limited number of available studies precluded subgroup analyses. All between‐group models without outliers are displayed in Table 3.

**Table 3:** Between‐group effects of LTPP on clinical outcome excluding outliers.

Outcome	*K* (no. effects)	Satt df^a^	SMD^b^	NNT	95% CI	95% PI	*p*	Heterogeneity				
								Within‐study		Between‐study		Overall
								*τ* ^2^	*I* ^2^ (%)	*τ* ^2^	*I* ^2^ (%)	*I* ^2^ (%)
**Severity of anxiety**												
Across all trials^c^												
End of treatment	6 (13)	4.04	0.13	14	−0.19, 0.44	−0.45, 0.70	0.33	0.005	7.52	0.025	37.49	45.01
End of treatment—high RoB studies removed^d^	4 (10)	—	0.33	6	0.05, 0.62	0.05, 0.62	0.03 ^∗^	—	—	—	—	0.00
Follow‐up (3–6 months)	2 (4)	—	0.42	5	−2.78, 3.62	−2.78, 3.62	0.35	—	—	—	—	0.00
Follow‐up (12–18 months)	2 (5)	—	0.45	4	−0.90, 1.79	−0.90, 1.79	0.15	—	—	—	—	0.00
All time‐points	6 (31)	—	0.09	20	−0.21, 0.40	−0.48, 0.66	0.47	—	—	—	—	45.41
All time‐points—high RoB studies removed^d^	4 (27)	—	0.24	8	−0.09, 0.57	−0.18, 0.66	0.10	—	—	—	—	13.14
Versus active control^c^												
End of treatment	3 (6)	—	0.31	6	−0.77, 1.38	−1.55, 2.16	0.35	—	—	—	—	66.87
All time‐points	3 (10)	—	0.05	33	−0.81, 0.92	−1.33, 1.44	0.81	—	—	—	—	52.35
Versus active treatment^c^												
End of treatment	4 (10)	—	0.13	14	−0.39, 0.65	−0.76, 1.02	0.48	—	—	—	—	49.93
End of treatment—high RoB studies removed^d^	3 (8)	—	0.27	7	−0.28, 0.83	−0.47, 1.02	0.17	—	—	—	—	20.75
All time‐points	4 (27)	—	0.06	30	−0.43, 0.55	−0.77, 0.89	0.73	—	—	—	—	47.32
High RoB studies removed^d^	3 (25)	—	0.14	13	−0.56, 0.83	−0.92, 1.20	0.48	—	—	—	—	41.57
**Global distress**												
Across all trials^c^												
End of treatment	10 (23)	7.72	0.32	6	0.14, 0.50	−0.10, 0.74	0.003 ^∗∗^	0.018	24.95	0.009	12.62	37.57
End of treatment—high RoB studies removed^d^	8 (20)	6.20	0.35	6	0.13, 0.57	−0.14, 0.84	0.008 ^∗∗^	0.011	13.73	0.022	29.18	42.91
Follow‐up (3–6 months)	3 (7)	—	0.41	5	−0.20, 1.01	−0.20, 1.01	0.10	—	—	—	—	0.00
Follow‐up (12–18 months)	3 (7)	—	0.39	5	0.01, 0.78	0.01, 0.78	0.05 ^∗^	—	—	—	—	0.00
Follow‐up (24–36 months)	2 (4)	—	0.48	4	−1.75, 2.70	−2.92, 3.88	0.22	—	—	—	—	65.83
All time‐points	10 (43)	7.16	0.33	6	0.15, 0.50	−0.10, 0.75	0.003 ^∗∗^	0.017	26.43	0.009	14.30	40.73
All time‐points—high RoB studies removed^d^	8 (40)	5.85	0.36	5	0.14, 0.57	−0.14, 0.85	0.007 ^∗∗^	0.015	21.36	0.018	24.71	46.07
Versus active control^c^												
End of treatment	4 (8)	—	0.39	5	−0.04, 0.82	−0.27, 1.05	0.06	—	—	—	—	33.82
End of treatment—high RoB studies removed^d^	2 (4)	—	0.55	4	−1.34, 2.45	−1.51, 2.61	0.17	—	—	—	—	8.17
All time‐points	4 (19)	—	0.25	8	−0.48, 0.98	−1.22, 1.72	0.36	—	—	—	—	77.86
All time‐points—high RoB studies removed^d^	2 (10)	—	0.55	4	−1.30, 2.39	−1.46, 2.56	0.17	—	—	—	—	8.06
Versus active treatment^c^												
End of treatment	7 (17)	5.02	0.27	7	0.02, 0.52	−0.22, 0.77	0.037 ^∗^	0.01	16.42	0.02	21.68	38.10
End of treatment—high RoB studies removed^d^	6 (16)	4.34	0.28	7	−0.01, 0.57	−0.30, 0.86	0.057	0.01	15.19	0.02	29.20	44.39
All time‐points	7 (31)	4.60	0.27	7	0.04, 0.51	−0.21, 0.76	0.032 ^∗^	0.01	21.32	0.01	17.66	38.98
All time‐points—high RoB studies removed^d^	6 (30)	4.11	0.28	7	0, 0.56	−0.29, 0.85	0.050	0.01	19.30	0.02	26.13	45.44
**Clinical events**												
Across all trials^c^												
End of treatment	3 (13)	—	0.32	6	−0.56, 1.21	−1.13, 1.77	0.26	—	—	—	—	59.51
End of treatment—high RoB studies removed^d^	2 (5)	—	0.54	3	−1.40, 2.49	−1.40, 2.49	0.18	—	—	—	—	0.00
All time‐points	3 (18)	—	0.33	6	−0.51, 1.16	−1.08, 1.73	0.24	—	—	—	—	65.92
All time‐points—high RoB studies removed^d^	2 (10)	—	0.52	4	−0.69, 1.73	−0.69, 1.73	0.12	—	—	—	—	0.00

*Note: K*, number of clusters (i.e., total number of effects within clusters); Satt df, Satterthwaite degrees of freedom; SMD, standardized mean difference, expressed as Hedges’s *g*; *τ* ^2^, variance component, where *τ* ^2^ (within‐study) represents the variance of true effects within clusters, and *τ* ^2^ (between‐study) represents between‐study heterogeneity; *I* ^2^, proportion of variance due to heterogeneity rather than sampling error, where *I* ^2^ (within‐study) represents within‐cluster heterogeneity, and *I* ^2^ (between‐study) represents between‐study heterogeneity.

Abbreviations: 95% CI, 95% confidence interval; 95% PI, 95% prediction interval; NNT, number needed to treat; RoB, risk of bias.

^a^Satt df value indicates a three‐level model in which variance is partitioned at the cluster level.

^b^Effect sizes have been reverse‐scored so that positive values favor LTPP.

^c^Bateman and Fonagy [70], Bastos et al. [77], and Walton et al. [82] were excluded as outliers from all‐trials models; Bastos et al. [77] was excluded from active control models; and Walton et al. [82] from active treatment models.

^d^Doering et al. [75] and Clarkin et al. [71] were excluded from all‐trials models due to high risk of bias; Bateman and Fonagy [70] and Doering et al. [75] from active control models; and Clarkin et al. [71] from active treatment models.

^∗^ *p*  < 0.05.

^∗∗^ *p*  < 0.01.

^∗∗∗^ *p*  < 0.001.

##### 3.6.1.1. Across All Trials

For anxiety, pooled effects across all trials at treatment completion were small and nonsignificant (*K* = 6, SMD = 0.13, 95% CI [–0.19, 0.44]), with moderate heterogeneity (*I* ^2^ = 45%). Aggregated analyses across all timepoints similarly yielded small nonsignificant effects (SMD = 0.09) with wide confidence and prediction intervals. Follow‐up estimates at 3–6 and 12–18 months were based on very small numbers of studies and were highly imprecise. Exclusion of high‐risk‐of‐bias studies increased the point estimate at treatment completion, but this pattern was not consistent across models.

For global distress, pooled analyses across all trials demonstrated statistically significant reductions favoring LTPP at treatment completion (*K* = 10, SMD = 0.32, 95% CI [0.14, 0.50]), with low‐to‐moderate heterogeneity (*I^2^* = 38%). Effects remained statistically significant when aggregated across all timepoints (SMD = 0.33). The exclusion of high‐risk‐of‐bias studies produced slightly larger estimates without altering the overall pattern of findings. Clinical events showed slightly larger point estimates but were characterized by wide confidence and prediction intervals and substantial heterogeneity.

#### 3.6.2. Subgroup Comparisons

##### 3.6.2.1. LTPP Versus Active Controls

For anxiety, effects relative to active controls were nonsignificant at treatment completion and across all timepoints, with wide confidence and prediction intervals and substantial heterogeneity. Removal of high‐risk‐of‐bias studies did not materially improve precision or alter conclusions. For global distress, effects at treatment completion were of moderate magnitude but did not reach statistical significance, and heterogeneity increased markedly when pooled across timepoints. Exclusion of high‐risk‐of‐bias studies attenuated estimates and increased imprecision, indicating the instability of effects within this subgroup.

##### 3.6.2.2. LTPP Versus Active Treatments

For anxiety, comparisons with active treatments yielded negligible and nonsignificant effects across all models, indicating no meaningful differences between LTPP and other active psychological interventions. For global distress, LTPP demonstrated small but statistically significant effects at treatment completion and when aggregated across all timepoints. These effects were broadly consistent with those observed for depressive symptom severity, although confidence and prediction intervals indicated variability in expected effects across future trials. Excluding high‐risk‐of‐bias studies modestly increased effect estimates without changing the substantive conclusions.

#### 3.6.3. Psychosocial Functioning

Table 4 presents between‐group effects comparing LTPP across all trials for psychosocial functioning, including interpersonal functioning and quality of life. Inconsistent reporting across trials limited statistical power, restricting quantitative synthesis and precluding follow‐up interval‐specific and subgroup analyses. Interpersonal functioning was the only outcome reported with sufficient frequency to permit multilevel modeling, yielding small, marginally non‐significant effects favoring LTPP. In contrast, effects on quality of life were equivocal, with point estimates close to zero and wide confidence and prediction intervals. Across models, heterogeneity was substantial, limiting confidence in the pooled estimates.

**Table 4:** Between‐group effects of LTPP on psychosocial functioning across all trials excluding outliers.

Outcome	*K* (no. effects)	Satt df	SMD	NNT	95% CI	95% PI	*p*	Heterogeneity					
								Within‐study		Between‐study			Overall
								*τ* ^2^	*I* ^2^ (%)	*τ* ^2^		*I* ^2^ (%)	*I* ^2^ (%)
Interpersonal functioning^c^													
End of treatment	7 (14)	5.52	0.31	6	−0.08, 0.71	−0.67, 1.29	0.10	0.028	17.32	0.102	63.68		80.99
End of treatment—high RoB studies removed^d^	6 (13)	4.59	0.36	5	−0.10, 0.82	−0.72, 1.44	0.10	0.028	16.83	0.108	65.65		82.48
All time‐points	7 (33)	5.41	0.29	7	−0.08, 0.67	−0.65, 1.23	0.10	0.032	22.67	0.084	59.32		81.99
All time‐points—high RoB studies removed^d^	6 (32)	4.53	0.33	6	−0.10, 0.77	−0.70, 1.37	0.10	0.032	21.36	0.094	62.04		83.40
Quality of life^c^													
End of treatment	5 (15)	—	0.18	11	−0.13, 0.48	−0.40, 0.70	0.18	—	—	—	—		40.60
All time‐points	5 (29)	—	0.15	12	−0.19, 0.49	−0.53, 0.83	0.28	—	—	—	—		65.98

*Note: K*, number of clusters (total number of effects within clusters); Satt df, Satterthwaite degrees of freedom; SMD, standardized mean difference, expressed as Hedges’s *g* values; *τ* ^2^, variance component, where *τ* ^2^ (within‐study) represents the variance of true effects within clusters and *τ* ^2^ (between‐study) represents between‐study heterogeneity; *I* ^2^, proportion of variance due to heterogeneity rather than sampling error, where *I* ^2^ (within‐study) represents within‐cluster heterogeneity and *I* ^2^ (between‐study) represents between‐study heterogeneity.  ^∗^ *p* < 0.05,  ^∗∗^ *p* < 0.01, and  ^∗∗∗^ *p* < 0.001 indicate statistical significance.

Abbreviations: 95% CI, 95% confidence interval; 95% PI, 95% prediction interval; NNT, number needed to treat; RoB, risk of bias.

^a^The presence of a Satt df value indicates a three‐level model in which variance can be partitioned at the cluster level.

^b^Effect sizes have been reverse‐scored so that positive values favor LTPP.

^c^Bateman and Fonagy [70], Bastos et al. [77], and Walton et al. [82] were excluded as outliers.

^d^Doering et al. [75] and Clarkin et al. [71] were excluded due to high risk of bias.

### 3.7. Within‐Group Effects

Although within‐group effects do not permit causal attribution, they remain useful as indicators of the magnitude and durability of clinical change, especially when between‐group effect sizes are modest or uncertain. Within‐group effects could be calculated for 14 of the 16 included studies; baseline data were unavailable in the Robinson et al. [79] and Kool et al. [84] trials. Table S2 summarizes within‐group changes in depression severity, anxiety severity, and global distress from baseline to post‐treatment, follow‐up intervals, and aggregated across all timepoints, following the removal of outliers.

Across all studies, LTPP demonstrated a very large within‐group reduction in depressive symptoms at treatment termination (*K* = 14, SMD = 1.45, 95% CI [0.87, 2.03], *p*  < 0.001). Heterogeneity was substantial and largely attributable to between‐study variance (93%), with minimal within‐study variability (5%). The prediction interval was wide (SMD = −0.76 to 3.66), indicating considerable variability in expected effects across future studies. Outlier analyses identified three studies [75, 77, 78], which were subsequently excluded from all within‐group models. Following their exclusion, the end‐of‐treatment within‐group effect remained statistically significant (*K* = 11, SMD = 1.33, 95% CI [1.01, 1.64], *p*  < 0.001), and statistically significant within‐group reductions were also observed across multiple follow‐up intervals, extending up to 3 years post‐treatment.

For anxiety symptoms, LTPP was associated with moderate within‐group reductions at treatment completion. Follow‐up analyses were limited by sparse data beyond 12–18 months; effects at this interval remained positive but imprecise, with wide confidence and prediction intervals. When aggregated across all available timepoints, pooled effects remained moderate, albeit with substantial heterogeneity. For global distress, large within‐group improvements were observed at treatment completion. Follow‐up analyses were again constrained by limited reporting, although improvements persisted at the 12–18‐month interval. Aggregated analyses across all timepoints yielded a moderate, statistically significant reduction in global distress, accompanied by substantial between‐study heterogeneity.

### 3.8. Publication Bias

Publication bias was evaluated using Egger Sandwich tests, standard Egger regressions, and 3PSM likelihood ratio tests, with detailed results provided in the Supporting Information. Analyses were conducted for all‐comparator models and subgroup comparisons (LTPP versus active treatments and LTPP versus active controls). Across outcomes and subgroup analyses, there was no significant evidence of publication bias, as indicated by nonsignificant standard error slopes and selection likelihood tests.

### 3.9. GRADE Assessment

Certainty of evidence ratings, following the GRADE criteria, were applied to pooled effects on three outcomes at treatment termination: depressive symptom severity (critical outcome), anxiety symptoms, and global distress. Ratings were based on pooled estimates derived from outlier‐excluded models, further validated through sensitivity analyses excluding high‐risk studies. Table 5 summarizes GRADE assessments across all‐trial models and subgroup conditions (LTPP versus active controls and LTPP versus active treatments), reflecting evidence quality and clinical relevance.

**Table 5:** Summary of findings for LTPP effects on clinical outcomes at end of treatment, excluding outliers and high‐risk‐of‐bias studies.

Outcome and follow‐up	Patients (studies), *N*	Absolute effects			Certainty	Reasons for downgrading	Interpretation
		Other treatments	LTPP	Difference (SMD, 95% CI)			
Main analysis: LTPP vs. all trials							
Depression symptoms, self‐ and observer‐rated measures of depressive symptoms (critical)	1141 (11 RCTs)	—	—	**0.34** (0.20 to 0.47)	⨁⨁⨁⨁ High	No downgrading	Long‐term psychodynamic psychotherapy results in a slight decrease in depression symptoms compared to other treatments.
Anxiety symptoms, self‐ and observer‐rated measures of anxiety symptoms	475 (4 RCTs)	—	—	**0.33** (0.05 to 0.62)	⨁⨁◯◯ Low	Very serious imprecision	Long‐term psychodynamic psychotherapy may result in a slight decrease in anxiety symptoms compared to other treatments.
Global distress, self‐reported measures of global distress	1005 (8 RCTs)	—	—	**0.35** (0.13 to 0.57)	⨁⨁◯◯ Low	Very serious imprecision	Long‐term psychodynamic psychotherapy may result in a slight decrease in global distress compared to other treatments.
Subgroup: LTPP vs. active control							
Depression symptoms, self‐ and observer‐rated measures of depressive symptoms (critical)	238 (4 RCTs)	—	—	**0.61** (−0.03 to 1.25)	⨁◯◯◯ Very low	Extremely serious imprecision	The evidence is very uncertain about the effect of long‐term psychodynamic psychotherapy on depression symptoms when compared to active controls.
Anxiety symptoms, self‐ and observer‐rated measures of anxiety symptoms	202^a^ (3 RCTs)	—	—	**0.31** (−0.77 to 1.38)	⨁◯◯◯ Very low	Serious risk of bias; extremely serious imprecision	The evidence is very uncertain about the effect of long‐term psychodynamic psychotherapy on anxiety symptoms when compared to active controls.
Global distress, self‐reported measures of global distress	189 (2 RCTs)	—	—	**0.55** (−1.34 to 2.45)	⨁◯◯◯ Very low	Extremely serious imprecision	The evidence is very uncertain about the effect of long‐term psychodynamic psychotherapy on global distress when compared to active controls.
Subgroup: LTPP vs. active treatment							
Depression symptoms, self‐ and observer‐rated measures of depressive symptoms (critical)	903 (7 RCTs)	—	—	**0.27** (0.10 to 0.45)	⨁⨁⨁◯ Moderate	Serious imprecision	Long‐term psychodynamic psychotherapy probably slightly decreases depression symptoms when compared to active treatments.
Anxiety symptoms, self‐ and observer‐rated measures of anxiety symptoms	415 (3 RCTs)	—	—	**0.27** (−0.28 to 0.83)	⨁◯◯◯ Very low	Extremely serious imprecision	The evidence is very uncertain about the effect of long‐term psychodynamic psychotherapy on anxiety symptoms when compared to active treatments.
Global distress, self‐reported measures of global distress	816 (6 RCTs)	—	—	**0.28** (−0.01 to 0.57)	⨁⨁◯◯ Low	Very serious imprecision	Long‐term psychodynamic psychotherapy may result in a slight decrease in global distress when compared to active treatments.

^a^Estimate based on the inclusion of two studies with a high risk of bias [70, 75].

#### 3.9.1. Across All Trials

The certainty of evidence for depressive symptom severity was rated as high, while for global distress and anxiety symptoms it was rated as low. No serious issues regarding inconsistency, indirectness, or publication bias were identified, and all three outcome estimates reached statistical significance. Across trials, no serious concerns regarding the risk of bias were evident, with over 50% of studies rated low‐risk for allocation concealment, assessor blinding, and completeness of outcome data.

Imprecision was assessed using the partially contextualized confidence‐interval GRADE approach. In the absence of established specific thresholds for each outcome, SMDs of ±0.20, ±0.50, and ±0.80 were used as guiding thresholds for small, moderate, and large effects, respectively; certainty was downgraded by one, two, or three levels according to the number of thresholds crossed by the 95% CI. The null value was not treated as an additional threshold. Optimal information size (OIS) was examined as a secondary indicator of precision. OIS—the minimum required sample size to power a small effect of SMD 0.20 [64]— was calculated using G^ ∗^ Power Version 3.1.9.6 [86], with *α* set at 0.05 and power at 0.8. The resulting OIS was 788 participants. Sample sizes for depressive symptoms (*N* = 1141) and global distress (*N* = 1005) but not anxiety symptoms (*N* = 475) exceeded this threshold, indicating sufficient statistical power. The lower bound of 95% CIs as true effects indicated two‐level imprecision downgrading for anxiety and global distress outcomes, given that their lower bounds crossed the small and moderate thresholds (SMD = 0.20 and 0.50).

##### 3.9.1.1. Updated Criteria for ESTs

Given that evidence quality for the primary outcome (severity of depression) was high, and the pooled effect surpassed the MID threshold (SMD ≥ 0.24) for clinical relevance, LTPP warrants a Strong recommendation when compared across all trials. However, the absence of clinically relevant improvements in functional outcomes currently precludes assigning a Very Strong recommendation.

#### 3.9.2. Active Controls Subgroup

For comparisons with active controls, the certainty of evidence was rated as very low across depressive symptoms, anxiety symptoms, and global distress outcomes. These estimates were derived from pooled effects after excluding high‐risk‐of‐bias studies. However, the anxiety estimate was limited by having only three contributing studies [70, 74, 75], with two of these [70, 75] classified as high‐risk and thereby preventing estimation after their removal.

Across outcomes, no serious concerns regarding indirectness or publication bias emerged. Nevertheless, serious concerns arose regarding the risk of bias for anxiety outcomes, prompting a downgrade. There were serious concerns regarding imprecision across all three outcomes: the confidence interval for depressive symptoms encompassed effects ranging from trivial to large benefit, while those for anxiety symptoms and global distress encompassed clinically important effects favouring either LTPP or the active control. Each interval crossed at least three pre‐specified effect‐magnitude thresholds, resulting in three‐level downgrades for imprecision.

##### 3.9.2.1. Updated Criteria for ESTs

LTPP demonstrated clinically meaningful but nonsignificant differences compared to active controls. Given the overall very low certainty of the evidence, a Weak recommendation is most appropriate in this subgroup.

#### 3.9.3. Active Treatments Subgroup

Within the active treatments subgroup, the certainty of evidence was rated as moderate for depressive symptoms, low for global distress, and very low for anxiety outcomes. Across these outcomes, no serious concerns emerged regarding indirectness, publication bias, or the risk of bias. For depressive symptoms, the 95% CI crossed the small‐effect threshold, resulting in a one‐level downgrade for imprecision. For global distress, the CI crossed the small‐ and moderate‐effect thresholds, resulting in a two‐level downgrade. For anxiety symptoms, the CI encompassed effects ranging from a small effect favouring active treatment to a large effect favouring LTPP and crossed at least three thresholds, resulting in a three‐level downgrade. The OIS was reached for depressive symptoms and global distress but not for anxiety symptoms, supporting the fragility of the anxiety estimate.

##### 3.9.3.1. Updated Criteria for ESTs

Given moderate‐quality evidence demonstrating a clinically meaningful effect exceeding the MID for depressive symptoms, LTPP warrants a Strong recommendation compared to active treatments for treating depression.

## 4. Discussion

This meta‐analysis synthesizes the current evidence regarding the efficacy of LTPP in reducing depressive symptoms. According to updated EST criteria, we found that LTPP warrants a Strong recommendation for the treatment of depression symptoms when compared across all trials and when compared with active treatments specifically, based on moderate‐ to high‐quality evidence demonstrating clinically meaningful effects. These findings corroborate recent Strong recommendations for psychodynamic treatments outlined by a recent umbrella review [53]. Comparisons with active control conditions support only a Weak recommendation owing to lower certainty and imprecision in the available evidence.

The included RCTs exhibited considerable variation in psychodynamic approach, treatment duration, patient populations, and comparator interventions, highlighting the breadth and flexibility of LTPP in clinical practice. By integrating RCT data across patients diagnosed with depression and/or personality disorders, this study aligns with recent calls for more transdiagnostic research approaches [48, 87]. Such inclusion enhances generalizability, mitigating the limitations imposed by narrowly defined diagnostic categories that fail to reflect real‐world clinical complexity.

Interventions were implemented in a range of service contexts, including outpatient psychotherapy clinics, specialist PD services, and day‐hospital programs. Treatment formats likewise differed, with some models delivered exclusively as individual psychotherapy (e.g., TFP and psychoanalysis), while others combined individual and group‐based components (e.g., MBT programs). Session frequency also varied substantially, ranging from once‐weekly psychotherapy to higher‐frequency analytic treatments or program‐based day‐hospital interventions. While these differences reflect the diversity with which long‐term psychodynamic approaches are implemented in routine clinical practice, the relatively small number of available trials precluded formal examination of whether treatment setting, format, or intensity moderated outcomes. Importantly, despite methodological variability among the trials, low between‐study heterogeneity in depression outcomes suggests that broader inclusion criteria did not introduce substantial measurement inconsistencies.

Within‐group comparisons exhibited large effects for reducing depressive symptoms and global distress at treatment completion and across follow‐up up to 36‐months post‐treatment, suggesting that LTPP is associated with sustained symptomatic improvement. Furthermore, between‐group summary effect estimates indicated that LTPP yields small but clinically meaningful (SMD >0.24) reductions in depressive symptoms and global distress at treatment completion compared with all comparators, with benefits also broadly sustained across subsequent follow‐up periods. These results concur with previous meta‐analyses indicating that LTPP is associated with modestly greater improvement in psychiatric symptoms and target problems compared to less intensive or alternative psychotherapies in individuals with complex mental disorders [39, 43].

Given the absence of robust, empirically validated thresholds for clinically meaningful differences between active interventions in depression, we adopted the threshold of SMD ±0.24 proposed by Cuijpers et al. [64]. This threshold, derived from patient‐anchored utility data and originally proposed to identify MIDs in treatment‐versus‐control contrasts, has been conservatively applied here to evaluate the potential clinical relevance of LTPP when compared with both active treatments and active control conditions. While this extrapolation remains provisional, it offers a cautious benchmark in the absence of more robust condition‐specific MID estimates for depression.

Crucially, clinically meaningful effects were retained even in comparisons restricted to active, empirically supported comparators such as CBT, DBT, or SCM. The relatively narrow CIs surrounding the pooled effect for depression severity in this subgroup underscore the precision and reliability of the current evidence for LTPP as a treatment for depression.

As SMDs are difficult to interpret [88] and frequently misinterpreted in clinical settings [89], in non‐pre‐registered analyses, we additionally calculated NNT values using the method proposed by Kraemer and Kupfer [60]. The estimated NNTs were 7 for comparisons with active treatments, suggesting that LTPP must be delivered to roughly three or seven patients for one to benefit beyond the comparator. While this approach is methodologically conservative and facilitates interpretability in the absence of dichotomous data, it is not anchored to clinically meaningful thresholds such as the treatment response or remission. Future trials should prioritize the reporting of binary outcomes, including response and remission rates, to enhance clinical relevance and applicability.

Benefits associated with LTPP may be driven by change processes thought to be specific to psychodynamic approaches, including insight and improved mentalizing, which have been more robustly demonstrated in psychodynamic treatments than in other psychotherapy modalities [90, 91]. Current evidence in this area is promising; however, more process‐outcome data from well‐designed RCTs are needed to draw conclusions regarding how mechanisms of change in PDT differ from other well‐validated psychotherapies. For instance, future trials should include psychodynamic process measures, such as the Reflective Functioning Questionnaire [92] or Insight into Conflictual Relationship Patterns Scale [93], as well as measures assessing common factors active in psychotherapy, such as the quality of the therapeutic alliance, and process measures rooted in other theoretical approaches.

For outcome domains other than depression severity and global distress, effect estimates were more inconsistent and often inadequately powered, preventing clear interpretation. In particular, data on the impact of LTPP on quality of life and clinically important events (e.g., hospitalizations and suicide attempts) were insufficient and inconclusive, highlighting a critical gap for future research to address. Interpersonal functioning showed small yet markedly variable effects, consistent with findings from another meta‐analysis of LTPP RCTs [43], which similarly reported modest and heterogeneous effects in this domain. The limited number of contributing studies prevented detailed subgroup analysis to clarify this variability. In addition, substantial between‐study variance suggests possible moderating factors. Given PDT’s explicit emphasis on relational dynamics, the inconsistent effects on the interpersonal outcomes were unexpected, underscoring the need for further investigation.

To our knowledge, this meta‐analysis is the first systematic examination of the effects of LTPP for depression and is the first to apply contemporary EST criteria to evaluate its efficacy. Methodological strengths further reinforce the validity of our findings. Specifically, our approach utilized the GRADE framework to integrate considerations of bias, clinical significance, statistical power, and heterogeneity into a coherent quality assessment. Additionally, multilevel modeling was employed to robustly account for within‐study variance, minimizing misattribution of effects to between‐study heterogeneity. A key strength of this meta‐analysis is the sufficient number of studies (*K* = 16), allowing meaningful differentiation between LTPP effects relative to active treatments and other control conditions. Lastly, while some trials in this review were conducted under heavily controlled conditions, others (e.g., [76, 78, 84]) adopted more pragmatic designs, featuring broad inclusion criteria, allowance for concurrent medication, and delivery within routine care settings, strengthening the generalizability of our findings to routine care.

### 4.1. Limitations and Future Directions

Despite these strengths, several limitations warrant consideration. First, the relatively small number and heterogeneity of the included RCTs constrained the scope and depth of conclusions. Although robust end‐of‐treatment effects for depressive symptoms were identified, the limited data prevented meaningful moderator analyses of potential moderating variables. This significantly constrains the clinical interpretability of the evidence, as it precludes meaningful testing of potentially important moderators amidst a highly heterogeneous set of studies. As more studies accumulate, future meta‐analyses must test for potential moderating variables across complexity dimensions, including baseline severity, illness chronicity, comorbid personality pathology, trauma histories, and interpersonal dysfunction, as well as treatment‐related factors such as duration, intensity, and delivery format. Without this information, it remains difficult for clinicians and policymakers to provide tailored, evidence‐based recommendations for when LTPP is most likely to be effective.

A key consideration concerns the operationalization of complex depression. While the construct informed study inclusion on the basis of clinical complexity, there remains limited consensus regarding how such a subgroup should be defined and measured, with variability in how complexity is indexed across studies. Nonetheless, focusing on complex presentations is warranted, given both the high prevalence of depression and consistent evidence that comorbidity, chronicity, and personality‐related difficulties are associated with a poorer response to brief, symptom‐focused interventions and may benefit from longer‐term, relationally oriented approaches. Without such a framing, efforts to synthesize and interpret treatment effects for this prevalent subset risk being unproductive. Future work should therefore refine and operationalize the construct in clinically actionable terms. In particular, there is a need to develop and validate assessment strategies that delineate subgroups at the level of treatment stratification to better identify individuals who may benefit from longer‐term interventions, including LTPP.

In addition, a lack of trials prevented us from exploring the variability between specific LTPP approaches. Consequently, LTPP was analyzed as a unified intervention without fully accounting for potential variability between specific psychodynamic approaches. This may be problematic because although LTPP represents a unified therapeutic approach, differences in emphasis and technique may conceal meaningful and clinically useful differences.

Subgroup analyses of LTPP versus control conditions yielded greater imprecision and inconsistency, primarily reflecting the small subgroup size (*K* = 6) and variability in defining active control conditions. For example, one study [78] employed a pragmatic active control involving any UK National Institute for Health and Care Excellence (NICE)‐recommended treatment for treatment‐resistant depression (including psychotherapy and pharmacotherapy), whereas another study [76] utilized 6 months of supportive therapy as an active comparator. Such heterogeneity makes it challenging to clearly establish LTPP’s relative effectiveness compared to that of active controls. Although moderate to large effects emerged, the considerable uncertainty in estimates limited the strength of clinical recommendations (i.e., a Weak recommendation according to the EST criteria). Further trials clarifying active control definitions and expanding subgroup comparisons are required for more robust conclusions regarding LTPP’s effectiveness compared with active controls.

A key limitation was our inability to model psychotropic medication use due to inconsistent reporting and lack of individual‐level data: although 10/16 trials reported arm‐specific baseline rates, sparse information on medication class, dose, and trajectory precluded covariate adjustment. Comparable baseline medication use and robust effects in trials with strict pharmacotherapy protocols suggest that LTPP’s benefits extend beyond those of drug treatment. However, future RCTs should longitudinally track and share individual‐level medication data to statistically isolate the effects of pharmacotherapy and psychotherapy.

Moreover, fewer than 10 of the 16 trials included follow‐up data, restricting reliable conclusions regarding sustained treatment efficacy. Echoing Woll and Schönbrodt ([43], p. 66), we emphasize the critical importance of extensive follow‐up data: “For a discipline assuming that treatment effects carry on and even continue to improve after the treatment finishes, more extensive follow‐up data should be provided in the future.”

Another notable gap is the limited reporting of secondary outcomes, including hospital utilization, suicide attempts, and parasuicidal behaviors, which are essential indicators of real‐world clinical impact. Likewise, quality‐of‐life data remain surprisingly sparse given the psychodynamic emphasis on long‐term functional recovery beyond mere symptomatic relief. A parsimonious quality‐of‐life measure, such as the EQ‐5D, should be routinely implemented to enable a more holistic evaluation of life satisfaction in the face of ongoing symptomatology, as well as to enable more cost‐effectiveness analyses.

Cost‐effectiveness evaluations of LTPP remain insufficiently addressed. Given the resource‐intensive nature of LTPP, clear evidence demonstrating economic viability is crucial. Only three trials in this review included economic analyses, leaving the cost–benefit balance largely unexplored [94–96]. In the Helsinki Psychotherapy study [61], LTPP produced somewhat greater long‐term symptom improvement but incurred substantially higher direct costs than shorter‐term therapies, resulting in unfavorable cost‐effectiveness estimates over 5 years [96]. Similarly, in the Tavistock Adult Depression Study [78], a trial of LTPP for treatment‐resistant depression, modest gains in quality‐adjusted life years exceeded the current NICE willingness‐to‐pay thresholds [95]. Lastly, in a trial of mentalization‐based day hospital treatment for BPD [80], remission rates were higher, but the intervention was not cost‐effective compared to another active treatment (i.e., specialist TAU) when evaluated using quality‐adjusted life years [94]. Overall, more research is needed concerning the cost‐effectiveness of LTPP, and longer follow‐up may be needed to capture potential downstream benefits such as reduced healthcare utilization. If clinical gains remain modest relative to cost, the scalability of LTPP within resource‐constrained health systems is uncertain. Future trials should therefore incorporate rigorous economic evaluations to determine whether its therapeutic benefits justify its opportunity cost.

The risk‐of‐bias assessment indicated that 75% of included studies presented “some concerns,” and 25% had a high risk of bias. Although comparable with other psychotherapy meta‐analyses [4], these findings highlight the critical need for more rigorous RCT designs. Future research should also explicitly control for researcher allegiance effects, which can substantially influence outcomes and bias efficacy estimates [97]. To do this, future trials should deploy clearer protocols that ensure external randomization and blinded outcome assessment, implement intensity‐matched, modality‐specific therapist training and supervision, avoid therapist crossover between conditions, and report all allegiance control measures explicitly in trial publications.

Additionally, although our inclusion criteria required that all treatment arms report mean depression scores exceeding validated clinical thresholds, it is possible that some samples were skewed by individuals with particularly severe symptoms, thereby inflating group averages and allowing the inclusion of patients who may not have met clinical criteria for depression. This is a limitation insofar as it introduces diagnostic heterogeneity, potentially diluting the precision of inferences drawn specifically about LTPP as a treatment for clinical depression. We have therefore cautiously interpreted these data as evidence that LTPP is effective in reducing depression severity, rather than as definitive support for its use in diagnosed depression. However, our sensitivity analysis—restricted to trials in which all participants were diagnosed using structured clinical interviews—suggests that LTPP is an effective treatment for diagnostically confirmed depressive samples, thereby mitigating concerns about diagnostic heterogeneity.

Another significant limitation of the current analysis is its limited global representation. All but two studies were conducted in Europe or predominantly white, English‐speaking contexts, thereby restricting cross‐cultural generalizability. To enhance global clinical relevance, future research should prioritize international collaboration, recruit more culturally and geographically diverse samples, and adapt interventions to ensure contextual sensitivity and feasibility across settings.

Given the growing evidence base for LTPP, future research should consider conducting noninferiority trials relative to other established treatments [98]. While superiority trials seek to demonstrate significant advantages of one intervention over another, noninferiority trials determine whether an intervention is not meaningfully less effective than an established comparator within a predefined margin. Given substantial evidence demonstrating comparable efficacy across psychotherapy modalities [2, 3, 99], research priorities should shift toward patient preference, acceptability, and adherence rather than solely efficacy.

## 5. Conclusion

This meta‐analysis provides evidence that LTPP is an EST for reducing depressive symptoms, including in cases of severe, chronic, and complex presentations. This finding is clinically significant given the disproportionate burden of illness, high rates of treatment resistance, and substantial resource utilization associated with complex depression. LTPP demonstrated small, clinically meaningful effects on depressive symptoms and global distress relative to active treatments, while comparisons with active controls produced larger but less certain estimates. The inclusion of pragmatic trials such as the Tavistock Adult Depression Study [78], alongside other trials incorporating naturalistic treatment elements, support the applicability of these findings to routine clinical practice.

However, notable gaps remain. Further research is needed on long‐term follow‐up outcomes, functional outcomes, cost‐effectiveness evaluations, and secondary clinical outcomes such as hospital utilization and suicide‐related behaviors. Addressing these knowledge gaps is critical to investigate LTPP’s potential as a treatment option for patients with depression, including those with more complex presentations.

## Funding

This research was funded by the Royal Australian and New Zealand College of Psychiatrists.

## Conflicts of Interest

The authors declare no conflicts of interest.

## Supporting Information

Additional supporting information can be found online in the Supporting Information section.

## Supporting information


**Supporting Information** The supporting information file includes the PRISMA 2020 checklist, details of effect size conversion methods, procedures for operationalizing end of treatment when treatment lengths differed, full within‐group effects analyses, and publication bias analyses.

## Data Availability

The data that support the findings of this study are openly available in the “Repository for Long‐Term Psychodynamic Psychotherapy for Complex Depression: A Systematic Review and Meta‐Analysis of Randomized Controlled Trials” at https://osf.io/85p4q/.
