# Supplementary material for: Long‐Term Psychodynamic Psychotherapy for Depression: A Systematic Review and Meta‐Analysis of Randomized Controlled Trials
Source: Depress Anxiety. 2026 Sep 23;2026:6172409. doi: 10.1155/da/6172409 (PMC13601849; doi:10.1155/da/6172409)
Supplement: Supplementary file 1 [file DA-2026-6172409-s001.docx]

# Supplementary materials for the manuscript entitled: “Long-Term Psychodynamic Psychotherapy for Depression: A Systematic Review and Meta-Analysis of Randomized Controlled Trials”

**Table S1**

*PRISMA 2020 checklist*

| **Topic** | **No.** | **Item** | **Location where item is reported** |
| --- | --- | --- | --- |
| **TITLE** |  |  |  |
| **Title** | 1 | Identify the report as a systematic review. | Title Page |
| **ABSTRACT** |  |  |  |
| **Abstract** | 2 | See the PRISMA 2020 for Abstracts checklist |  |
| **INTRODUCTION** |  |  |  |
| **Rationale** | 3 | Describe the rationale for the review in the context of existing knowledge. | Introduction, |
| **Objectives** | 4 | Provide an explicit statement of the objective(s) or question(s) the review addresses. | Introduction, |
| **METHODS** |  |  |  |
| **Eligibility criteria** | 5 | Specify the inclusion and exclusion criteria for the review and how studies were grouped for the syntheses. | Selection Criteria subsection |
| **Information sources** | 6 | Specify all databases, registers, websites, organisations, reference lists and other sources searched or consulted to identify studies. Specify the date when each source was last searched or consulted. | Search Strategy subsection |
| **Search strategy** | 7 | Present the full search strategies for all databases, registers and websites, including any filters and limits used. | OSF |
| **Selection process** | 8 | Specify the methods used to decide whether a study met the inclusion criteria of the review, including how many reviewers screened each record and each report retrieved, whether they worked independently, and if applicable, details of automation tools used in the process. | Data Extraction and risk of bias assessment subsection |
| **Data collection process** | 9 | Specify the methods used to collect data from reports, including how many reviewers collected data from each report, whether they worked independently, any processes for obtaining or confirming data from study investigators, and if applicable, details of automation tools used in the process. | Data Extraction and risk of bias assessment subsection |
| **Data items** | 10a | List and define all outcomes for which data were sought. Specify whether all results that were compatible with each outcome domain in each study were sought (e.g. for all measures, time points, analyses), and if not, the methods used to decide which results to collect. | Outcomes subsection |
|  | 10b | List and define all other variables for which data were sought (e.g. participant and intervention characteristics, funding sources). Describe any assumptions made about any missing or unclear information. | Outcomes subsection |
| **Study risk of bias assessment** | 11 | Specify the methods used to assess risk of bias in the included studies, including details of the tool(s) used, how many reviewers assessed each study and whether they worked independently, and if applicable, details of automation tools used in the process. | Data Extraction and risk of bias assessment subsection |
| **Effect measures** | 12 | Specify for each outcome the effect measure(s) (e.g. risk ratio, mean difference) used in the synthesis or presentation of results. | Statistical Analysis |
| **Synthesis methods** | 13a | Describe the processes used to decide which studies were eligible for each synthesis (e.g. tabulating the study intervention characteristics and comparing against the planned groups for each synthesis (item 5)). | Data Extraction and risk of bias assessment subsection |
|  | 13b | Describe any methods required to prepare the data for presentation or synthesis, such as handling of missing summary statistics, or data conversions. | Stastistical Analysis, Supplementary Materials, Section 1 |
|  | 13c | Describe any methods used to tabulate or visually display results of individual studies and syntheses. | Stastistical Analysis, |
|  | 13d | Describe any methods used to synthesize results and provide a rationale for the choice(s). If meta-analysis was performed, describe the model(s), method(s) to identify the presence and extent of statistical heterogeneity, and software package(s) used. | Stastistical Analysis, |
|  | 13e | Describe any methods used to explore possible causes of heterogeneity among study results (e.g. subgroup analysis, meta-regression). | Stastistical Analysis, |
|  | 13f | Describe any sensitivity analyses conducted to assess robustness of the synthesized results. | Stastistical Analysis, |
| **Reporting bias assessment** | 14 | Describe any methods used to assess risk of bias due to missing results in a synthesis (arising from reporting biases). | Statistical Analysis and Supplementary Section 3. |
| **Certainty assessment** | 15 | Describe any methods used to assess certainty (or confidence) in the body of evidence for an outcome. | Quality Assessment subsection |
| **RESULTS** |  |  |  |
| **Study selection** | 16a | Describe the results of the search and selection process, from the number of records identified in the search to the number of studies included in the review, ideally using a flow diagram. | Search Results subsection, and Figure 1 |
|  | 16b | Cite studies that might appear to meet the inclusion criteria, but which were excluded, and explain why they were excluded. | Search Results subsection |
| **Study characteristics** | 17 | Cite each included study and present its characteristics. | Table 1 |
| **Risk of bias in studies** | 18 | Present assessments of risk of bias for each included study. | Figure 2 |
| **Results of individual studies** | 19 | For all outcomes, present, for each study: (a) summary statistics for each group (where appropriate) and (b) an effect estimate and its precision (e.g. confidence/credible interval), ideally using structured tables or plots. | Figure 2, OSF Supplement |
| **Results of syntheses** | 20a | For each synthesis, briefly summarise the characteristics and risk of bias among contributing studies. | Each synthesis subsection |
|  | 20b | Present results of all statistical syntheses conducted. If meta-analysis was done, present for each the summary estimate and its precision (e.g. confidence/credible interval) and measures of statistical heterogeneity. If comparing groups, describe the direction of the effect. | Tables 2, 3, & 4 |
|  | 20c | Present results of all investigations of possible causes of heterogeneity among study results. | Tables 2, 3, & 4 |
|  | 20d | Present results of all sensitivity analyses conducted to assess the robustness of the synthesized results. | Tables 2, 3, & 4 |
| **Reporting biases** | 21 | Present assessments of risk of bias due to missing results (arising from reporting biases) for each synthesis assessed. | Supporting Information, Tables S3–S6 |
| **Certainty of evidence** | 22 | Present assessments of certainty (or confidence) in the body of evidence for each outcome assessed. | Table 5 |
| **DISCUSSION** |  |  |  |
| **Discussion** | 23a | Provide a general interpretation of the results in the context of other evidence. | Discussion |
|  | 23b | Discuss any limitations of the evidence included in the review. | Limitations and Recommendations subsection |
|  | 23c | Discuss any limitations of the review processes used. | Limitations and Recommendations subsection |
|  | 23d | Discuss implications of the results for practice, policy, and future research. | Limitations and Recommendations subsection |
| **OTHER INFORMATION** |  |  |  |
| **Registration and protocol** | 24a | Provide registration information for the review, including register name and registration number, or state that the review was not registered. | Methods |
|  | 24b | Indicate where the review protocol can be accessed, or state that a protocol was not prepared. | Methods |
|  | 24c | Describe and explain any amendments to information provided at registration or in the protocol. | Methods |
| **Support** | 25 | Describe sources of financial or non-financial support for the review, and the role of the funders or sponsors in the review. | Funding Information subsection |
| **Competing interests** | 26 | Declare any competing interests of review authors. | Competing Interests subsection |
| **Availability of data, code and other materials** | 27 | Report which of the following are publicly available and where they can be found: template data collection forms; data extracted from included studies; data used for all analyses; analytic code; any other materials used in the review. | Methods |

# PRISMA Abstract Checklist

| **Topic** | **No.** | **Item** | **Reported?** |
| --- | --- | --- | --- |
| **TITLE** |  |  |  |
| **Title** | 1 | Identify the report as a systematic review. | Yes |
| **BACKGROUND** |  |  |  |
| **Objectives** | 2 | Provide an explicit statement of the main objective(s) or question(s) the review addresses. | Yes |
| **METHODS** |  |  |  |
| **Eligibility criteria** | 3 | Specify the inclusion and exclusion criteria for the review. | Yes |
| **Information sources** | 4 | Specify the information sources (e.g. databases, registers) used to identify studies and the date when each was last searched. | Yes |
| **Risk of bias** | 5 | Specify the methods used to assess risk of bias in the included studies. | Yes |
| **Synthesis of results** | 6 | Specify the methods used to present and synthesize results. | Yes |
| **RESULTS** |  |  |  |
| **Included studies** | 7 | Give the total number of included studies and participants and summarise relevant characteristics of studies. | Yes |
| **Synthesis of results** | 8 | Present results for main outcomes, preferably indicating the number of included studies and participants for each. If meta-analysis was done, report the summary estimate and confidence/credible interval. If comparing groups, indicate the direction of the effect (i.e. which group is favoured). | Yes |
| **DISCUSSION** |  |  |  |
| **Limitations of evidence** | 9 | Provide a brief summary of the limitations of the evidence included in the review (e.g. study risk of bias, inconsistency and imprecision). | Yes |
| **Interpretation** | 10 | Provide a general interpretation of the results and important implications. | Yes |
| **OTHER** |  |  |  |
| **Funding** | 11 | Specify the primary source of funding for the review. | Yes |
| **Registration** | 12 | Provide the register name and registration number. | Yes |

*From:* Page MJ, McKenzie JE, Bossuyt PM, Boutron I, Hoffmann TC, Mulrow CD, et al. The PRISMA 2020 statement: an updated guideline for reporting systematic reviews. MetaArXiv. 2020, September 14. DOI: 10.31222/osf.io/v7gm2. For more information, visit: www.prisma-statement.org

**Section 1. Effect size conversion**

Chi-squared statistics were converted to Hedges’s g values using the ‘esc’ package version 0.5.1 in R (Lüdecke et al., 2019). Cohen’s d values were derived from Mann-Whitney U test results using the formula in Fritz et al. (2012) and from Pearson’s r and Fisher’s z statistics according to methods described by Borenstein et al. (2021). Proportions were transformed into log odds ratios and then to Cohen’s d, following the methods outlined by Harrer et al. (2021) and Borenstein et al. (2021). These Cohen’s d values were adjusted for small sample sizes and converted to Hedges’s g using the correction factor J (Hedges & Olkin, 2014).

## Section 2. Operationalising end of treatment when treatment lengths differ

The duration of treatment differed significantly between psychodynamic therapy (PDT) and non-PDT treatments in two studies (Knekt et al., 2008; Leuzinger-Bohleber et al., 2019). In the Knekt et al. (2008) trial, patients in the long-term psychodynamic psychotherapy (LTPP) arm received treatment for up to 36 months, with an average duration of 31.5 months. Conversely, patients in the short-term psychodynamic psychotherapy (STPP) and solution-focused therapy (SFT) arms were treated for averages of 5.7 and 7.5 months, respectively. Similarly, in the study by Leuzinger-Bohleber et al. (2019), patients undergoing psychoanalytic treatment (PAT) were offered treatment for up to 36 months, while those in the cognitive behavioural therapy (CBT) arm received treatment for up to 15 months.

The alignment of data collection time-points with the end of treatment varied, particularly for the shorter treatments. In the Knekt et al. (2008) study, outcomes were assessed at 7, 9, and 36 months post-treatment commencement. Leuzinger-Bohleber et al. (2019) conducted measurements at one, two, and three years after treatment began. This discrepancy in treatment length and data collection schedules presents challenges in comparing efficacy across different therapeutic approaches.

Discrepant treatment lengths within studies pose significant challenges, requiring reviewers to determine what constitutes the ‘end of treatment’ for each arm, often necessitating different end points for each treatment within the same study. In this meta-analysis, we defined 'end of treatment' as the closest available timepoint after all participants in the group had completed their treatment, or in the case of the Knekt et al. (2008) study, when treatment had ended on average.

For the Knekt trial, the 7 and 9-month timepoints were designated as the end of treatment for the STPP and SFT arms, respectively, with the 36-month timepoint serving as the end for the LTPP arm. According to Leichsenring & Rabung (2011), this approach likely introduces no bias as the effects observed between the 7 and 9-month evaluations are nearly identical. In the Leuzinger-Bohleber et al. (2019) study, even though the CBT treatment lasted a maximum of 15 months, the two-year mark was selected to represent the end of treatment for the CBT group.

Opting for different end-of-treatment timepoints for each group ensures that the evaluations reflect the most recent completion of treatment by all or most participants in the group. This is crucial, as data from ongoing treatments or follow-up timepoints do not reliably reflect treatment outcomes. Unfortunately, for the Doering et al. (2010) trial, it was not feasible to apply this method as the majority of patients in the PDT arm were still undergoing treatment at the final timepoint of measurement.

**Multi-arm trials**

Because three-level modelling was not always possible due to an insufficient number of clusters, two-level models were specified and within-study effects were aggregated to calculate single study-wide effects. To avoid double-counting participants in studies that compared more than two groups, descriptive statistics (mean, standard deviation, and sample size) of the two or more experimental (Clarkin et al. 2007) or control (Knekt et al., 2008; Kool et al., 2024) groups were pooled to create a single experimental or control group, respectively (Higgins et al., 2019). Pooling was performed using the ‘pool.groups’ function in the ‘dmetar’ package in R (Harrer et al., 2019).

**Table S2**

*Within-Group Effects of LTPP on Symptom Outcomes, Excluding Outliers*

| Outcome^b^ | K (no. effects) | Satt df^a^ | SMD | 95%CI | 95% PI | *p* | Heterogeneity | | | | |
| --- | --- | --- | --- | --- | --- | --- | --- | --- | --- | --- | --- |
|  |  |  |  |  |  |  | Within-study | | Between-study | | Overall |
|  |  |  |  |  |  |  | *τ²* | *I²* (%) | *τ²* | *I²* (%) | *I²* (%) |
| **Severity of Depression** |  |  |  |  |  |  |  |  |  |  |  |
| End of treatment | 11 (15) | 9.65 | 1.33 | 1.01, 1.64 | 0.31, 2.35 | < .001*** | 0.05 | 25.72 | 0.13 | 64.22 | 89.94% |
| High RoB studies removed^c^ | 9 (13) | 7.79 | 1.31 | 0.94, 1.68 | 0.20, 2.42 | < .001*** | 0.05 | 24.44 | 0.15 | 67.00 | 91.44% |
| Follow-up (3–6 months) | 2 (2) |  | 1.29 | -0.35, 2.93 | -0.35, 2.93 | .064 |  |  |  |  | 0.00% |
| Follow-up (12–18 months) | 4 (6) |  | 1.43 | 0.82, 2.04 | 0.21, 2.65 | .005** |  |  |  |  | 79.02% |
| Follow-up (24–36 months) | 2 (6) |  | 1.55 | -0.92, 4.02 | -2.22, 5.31 | .080 |  |  |  |  | 61.19% |
| All timepoints | 11 (35) | 9.02 | 1.22 | 1.04, 1.40 | 0.71, 1.73 | < .001*** | 0.01 | 12.22 | 0.04 | 57.29 | 69.51% |
| High RoB studies removed^c^ | 9 (31) | 7.44 | 1.19 | 0.99, 1.39 | 0.67, 1.71 | < .001*** | 0.00 | 6.98 | 0.04 | 63.68 | 70.66% |
| **Severity of Anxiety** |  |  |  |  |  |  |  |  |  |  |  |
| End of treatment | 5 (13) |  | 0.81 | 0.41, 1.21 | -0.09, 1.70 | .01* |  |  |  |  | 84.71% |
| High RoB studies removed^c^ | 3 (9) |  | 0.68 | 0.01, 1.35 | -0.58, 1.94 | .05* |  |  |  |  | 86.01% |
| Follow-up (12-18 months) | 2 (5) |  | 0.83 | -0.88, 2.53 | -1.88, 3.53 | .10 |  |  |  |  | 75.70% |
| All timepoints | 5 (39) |  | 0.90 | 0.42, 1.38 | -0.20, 2.01 | .01* |  |  |  |  | 93.84% |
| High RoB studies removed^c^ | 3 (31) |  | 0.73 | 0.24, 1.23 | -0.18, 1.65 | .02* |  |  |  |  | 86.20% |
| **Global Distress** |  |  |  |  |  |  |  |  |  |  |  |
| End of treatment | 7 (12) | 5.15 | 1.34 | 0.46, 2.22 | -1.91, 4.59 | .011* | 1.51 | 98.87 | 0.00 | 0.00 | 98.87% |
| High RoB studies removed^c^ | 5 (9) |  | 0.74 | 0.20, 1.27 | -0.53, 2.00 | .019* |  |  |  |  | 93.99% |
| Follow-up (12–18 months) | 2 (5) |  | 0.68 | -4.95, 6.31 | -9.00, 10.36 | .367 |  |  |  |  | 97.77% |
| All timepoints | 7 (29) |  | 0.74 | 0.40, 1.07 | -0.17, 1.64 | .002** |  |  |  |  | 93.00% |
| High RoB studies removed^c^ | 5 (21) |  | 0.76 | 0.27, 1.25 | -0.41, 1.93 | .013* |  |  |  |  | 95.30% |
|  | | | | | | | | | | | |

*Note.* K = number of clusters (total number of effects within clusters); Satt*(*df*)* = Satterthwaite degrees of freedom; SMD = standardized mean difference, expressed as Hedges’s *g* values; 95% CI = 95% confidence interval; 95% PI = 95% prediction interval; *τ²* = variance component, where *τ²* (within-study) represents the variance of true effects within clusters and *τ²* (between-study) represents between-study heterogeneity; *I²* = proportion of variance due to heterogeneity rather than sampling error, where *I²* (within-study) represents within-cluster heterogeneity and *I²* (between-study) represents between-study heterogeneity; RoB = Risk of Bias.

^a^ The presence of a Satt df value indicates a three-level model in which variance can be partitioned at the cluster level. ^b^ Effect sizes have been reverse-scored so that positive values indicate improvement.

^b^ Outlier analyses identified three studies (Fonagy et al., 2015; Doering et al., 2010; Bastos et al., 2015), which were subsequently excluded from all within-group models

^c^Bateman & Fonagy, 1999 and Clarkin et al. 2007, were removed due to being rated as high-risk-of-bias studies

**p* < .05, ***p* < .01, ****p* < .001.

## Section 3. Publication bias

The validity of pooled effects can be threatened by selective reporting that can occur when the availability of findings are influenced by outcome-dependent factors, such as statistical significance and magnitude of effect size (Dwan et al., 2013). This can occur at the between-study level, for instance when the decision or likelihood of a study being published hinges on the statistical significance of its findings – the so called ‘file-drawer problem’ (Scargle, 2000) - and at the within-study level where the decision to report only a subset of outcome data is made to increase publication chance. There are several techniques to examine the presence of publication bias however simulation studies have indicated the Egger Sandwich and three-parameter selection model (3PSM) likelihood ratio tests as the most appropriate for multilevel modelling (Rodgers & Pustejovsky, 2021).

As such, to test for publication bias, we conducted the Egger Sandwich test and 3PSM likelihood ratio test for three-level models, and standard Egger test and 3PSM likelihood ratio tests for two-level models. Notably, before conducting 3PSM tests for multi-level models, effect sizes need to be aggregated by study and outcome level as recommended by Rodgers & Pustejovsky (2021).

The Egger test assesses small-study effects in meta-analyses by regressing effect-size estimates on their standard errors. A significant non-zero slope indicates potential funnel-plot asymmetry, which may reflect publication bias (Egger et al., 1997). For multilevel models, cluster-robust inference was applied using the Egger Sandwich test (Rodgers & Pustejovsky, 2021). Similarly, the 3PSM model accounts for publication bias by jointly estimating the true effect size, between- study variance, and a selection function that models the likelihood of study inclusion based on *p*-values, thereby adjusting the effect size estimate to mitigate the influence of selective publication. If the 3PSM model significantly fits the data better than the null model, indicated by a significant likelihood ratio test, this suggests the presence of selective publication of significant results (McShane et al., 2016).

**Table S3**

*Assessment of Small-Study Effects Using Egger Regression Across All Trials*

| Outcome | Model | Test | *b* | *t* | *p* | 95% CI |
| --- | --- | --- | --- | --- | --- | --- |
| Severity of depression | End of treatment | Egger Sandwich test | 0.87 | 0.73 | 0.498 | −2.23, 3.97 |
| Severity of depression | End of treatment—high RoB studies removed | Egger Sandwich test | 0.76 | 0.62 | 0.568 | −2.64, 4.15 |
| Severity of depression | 3–6 months | Standard Egger regression | −1.36 | −0.58 | 0.623 | −11.50, 8.79 |
| Severity of depression | 12–18 months | Standard Egger regression | 0.42 | 0.39 | 0.72 | −2.94, 3.77 |
| Severity of depression | 24–36 months | Standard Egger regression | −0.04 | −0.01 | 0.995 | −74.81, 74.72 |
| Severity of depression | All time points | Egger Sandwich test | 0.8 | 0.75 | 0.487 | −1.97, 3.57 |
| Severity of depression | All time points—high RoB studies removed | Egger Sandwich test | 0.78 | 0.68 | 0.533 | −2.37, 3.93 |
| Severity of anxiety | End of treatment | Egger Sandwich test | −1.44 | −1.51 | 0.283 | −5.98, 3.09 |
| Severity of anxiety | End of treatment—high RoB studies removed | Standard Egger regression | −1.35 | −1.22 | 0.346 | −6.08, 3.39 |
| Severity of anxiety | All time points | Standard Egger regression | −1.71 | −1.35 | 0.248 | −5.24, 1.81 |
| Severity of anxiety | All time points—high RoB studies removed | Standard Egger regression | −1.39 | −1.06 | 0.402 | −7.03, 4.26 |
| Global distress | End of treatment | Egger Sandwich test | −0.10 | −0.10 | 0.93 | −3.43, 3.23 |
| Global distress | End of treatment—high RoB studies removed | Egger Sandwich test | −0.03 | −0.02 | 0.985 | −4.89, 4.84 |
| Global distress | 3–6 months | Standard Egger regression | −0.98 | −0.65 | 0.631 | −20.05, 18.09 |
| Global distress | 12–18 months | Standard Egger regression | −0.79 | −0.44 | 0.734 | −23.31, 21.74 |
| Global distress | All time points | Egger Sandwich test | 0.06 | 0.11 | 0.921 | −1.99, 2.12 |
| Global distress | All time points—high RoB studies removed | Egger Sandwich test | 0.16 | 0.23 | 0.837 | −2.62, 2.94 |
| Interpersonal functioning | End of treatment | Egger Sandwich test | −1.79 | −1.81 | 0.227 | −6.58, 3.00 |
| Interpersonal functioning | End of treatment—high RoB studies removed | Egger Sandwich test | −1.52 | −1.19 | 0.384 | −8.78, 5.74 |
| Interpersonal functioning | All time points | Egger Sandwich test | 0.51 | 0.3 | 0.793 | −6.21, 7.23 |
| Interpersonal functioning | All time points—high RoB studies removed | Egger Sandwich test | 0.83 | 0.38 | 0.74 | −8.75, 10.42 |
| Quality of life | End of treatment | Standard Egger regression | −1.07 | −0.65 | 0.563 | −6.34, 4.20 |
| Quality of life | End of treatment—high RoB studies removed | Standard Egger regression | −1.07 | −0.65 | 0.563 | −6.34, 4.20 |
| Quality of life | All time points | Standard Egger regression | −0.98 | −0.47 | 0.671 | −7.60, 5.65 |
| Quality of life | All time points—high RoB studies removed | Standard Egger regression | −0.98 | −0.47 | 0.671 | −7.60, 5.65 |
| Clinical events | End of treatment | Standard Egger regression | 0.61 | 0.27 | 0.832 | −28.13, 29.36 |
| Clinical events | All time points | Standard Egger regression | 0.02 | 0.01 | 0.992 | −24.25, 24.30 |

*Note.* Egger tests were conducted to examine small-study effects for each outcome and timepoint. Where robust variance estimation was used in the primary meta-analysis, the Egger sandwich test was applied; otherwise, the standard Egger regression test was used. The coefficient b represents the slope for study standard error. A statistically significant slope indicates potential funnel-plot asymmetry, which may reflect publication bias. Tests were repeated following removal of studies rated as high risk of bias (high RoB) where indicated. Confidence intervals are reported at the 95% level. Results should be interpreted cautiously in models with a small number of studies, as power to detect asymmetry is limited.

**Table S4**

*Three-Parameter Selection Model (3PSM) Analyses Across All Trials*

| Outcome | Timepoint | Bias Adjustment | α | *k* (*p* ≤ α) | δ | *LRT* | *p* |
| --- | --- | --- | --- | --- | --- | --- | --- |
| Severity of depression | End of treatment |  | 0.025 | 4 | 0.62 | 0.21 | .64 |
| Severity of depression | End of treatment | High RoB removed | 0.025 | 4 | 0.72 | 0.11 | .74 |
| Severity of depression | End of treatment |  | 0.05 | 4 | 0.38 | 1.16 | .28 |
| Severity of depression | End of treatment | High RoB removed | 0.05 | 4 | 0.36 | 1.22 | .27 |
| Severity of depression | 3–6 months |  | 0.025 | 2 | 0.17 | 1.24 | .27 |
| Severity of depression | 3–6 months |  | 0.05 | 2 | 0.51 | 0.20 | .65 |
| Severity of depression | 12–18 months |  | 0.025 | 0 | 99.96 | 5.20 | .02 |
| Severity of depression | 12–18 months |  | 0.05 | 0 | 5.79 | 1.10 | .29 |
| Severity of depression | 24–36 months |  | 0.025 | 2 | 0.45 | 0.16 | .69 |
| Severity of depression | 24–36 months |  | 0.05 | 2 | 1.21 | 0.01 | .93 |
| Severity of depression | All time points |  | 0.025 | 4 | 0.43 | 0.73 | .39 |
| Severity of depression | All time points | High RoB removed | 0.025 | 4 | 0.47 | 0.57 | .45 |
| Severity of depression | All time points |  | 0.05 | 4 | 0.59 | 0.29 | .59 |
| Severity of depression | All time points | High RoB removed | 0.05 | 4 | 0.57 | 0.35 | .55 |
| Severity of Anxiety | End of treatment |  | 0.025 | 1 | 0.54 | 0.15 | .70 |
| Severity of Anxiety | End of treatment | High RoB removed | 0.025 | 1 | 1.52 | 0.06 | .81 |
| Severity of Anxiety | End of treatment |  | 0.05 | 1 | 0.18 | 1.52 | .22 |
| Severity of Anxiety | End of treatment | High RoB removed | 0.05 | 1 | 0.32 | 0.56 | .45 |
| Severity of Anxiety | All time points |  | 0.025 | 1 | 0.29 | 0.57 | .45 |
| Severity of Anxiety | All time points | High RoB removed | 0.025 | 1 | 0.53 | 0.13 | .72 |
| Severity of Anxiety | All time points |  | 0.05 | 1 | 0.10 | 2.71 | .10 |
| Severity of Anxiety | All time points | High RoB removed | 0.05 | 1 | 0.11 | 1.88 | .17 |
| Global distress | End of treatment |  | 0.025 | 4 | 0.60 | 0.23 | .64 |
| Global distress | End of treatment | High RoB removed | 0.025 | 4 | 0.41 | 0.58 | .45 |
| Global distress | End of treatment |  | 0.05 | 4 | 1.75 | 0.29 | .59 |
| Global distress | End of treatment | High RoB removed | 0.05 | 4 | 1.14 | 0.01 | .91 |
| Global distress | 3–6 months |  | 0.025 | 1 | 1.45 | 0.04 | .85 |
| Global distress | 3–6 months |  | 0.05 | 1 | 4.18 | 0.54 | .46 |
| Global distress | 12–18 months |  | 0.025 | 2 | 0.52 | 0.13 | .72 |
| Global distress | 12–18 months |  | 0.05 | 2 | 1.28 | 0.02 | .89 |
| Global distress | All time points |  | 0.025 | 4 | 0.65 | 0.17 | .68 |
| Global distress | All time points | High RoB removed | 0.025 | 4 | 0.44 | 0.49 | .48 |
| Global distress | All time points |  | 0.05 | 4 | 0.63 | 0.20 | .66 |
| Global distress | All time points | High RoB removed | 0.05 | 4 | 0.30 | 1.02 | .31 |
| Clinical events | End of treatment |  | 0.025 | 1 | 1.37 | 0.02 | .88 |
| Clinical events | End of treatment |  | 0.05 | 1 | 3.53 | 0.39 | .53 |
| Clinical events | All time points |  | 0.025 | 1 | 45.05 | 0.97 | .32 |
| Clinical events | All time points |  | 0.05 | 1 | 100.00 | 2.19 | .14 |
| Interpersonal functioning | End of treatment |  | 0.025 | 3 | 0.41 | 0.49 | .48 |
| Interpersonal functioning | End of treatment | High RoB removed | 0.025 | 3 | 0.35 | 0.58 | .45 |
| Interpersonal functioning | End of treatment |  | 0.05 | 3 | 0.88 | 0.01 | .92 |
| Interpersonal functioning | End of treatment | High RoB removed | 0.05 | 3 | 0.74 | 0.05 | .83 |
| Interpersonal functioning | 12–18 months |  | 0.025 | 1 | 0.37 | 0.24 | .62 |
| Interpersonal functioning | 12–18 months |  | 0.05 | 1 | 1.09 | 0.00 | .96 |
| Interpersonal functioning | All time points |  | 0.025 | 2 | 1.30 | 0.04 | .84 |
| Interpersonal functioning | All time points | High RoB removed | 0.025 | 2 | 1.30 | 0.03 | .85 |
| Interpersonal functioning | All time points |  | 0.05 | 2 | 2.68 | 0.56 | .45 |
| Interpersonal functioning | All time points | High RoB removed | 0.05 | 2 | 2.59 | 0.47 | .49 |
| Quality of life | End of treatment |  | 0.025 | 1 | 0.86 | 0.01 | .93 |
| Quality of life | End of treatment |  | 0.05 | 1 | 0.23 | 0.98 | .32 |
| Quality of life | 12–18 months |  | 0.025 | 0 | 100.00 | 1.10 | .29 |
| Quality of life | 12–18 months |  | 0.05 | 0 | 0.06 | 1.75 | .19 |
| Quality of life | All time points |  | 0.025 | 1 | 0.74 | 0.03 | .86 |
| Quality of life | All time points |  | 0.05 | 1 | 0.16 | 1.41 | .23 |

*Note.* Results from three-parameter selection models (3PSM) using step-function weight functions with cut-points at α = .025 and α = .05. *k* (p ≤ α) denotes the number of effects within the lower significance interval. δ represents the estimated relative weight (selection parameter) for effects in the higher *p*-value interval relative to the lowest interval. *LRT* denotes the likelihood ratio test comparing the selection model with the no-selection model. “High RoB removed” indicates models re-estimated after excluding studies rated at high risk of bias. The depression 12–18-month model at α = .025 contained no study-level effects in the p ≤ α interval (k = 0) and produced an extreme δ estimate of 99.96. The nominally significant likelihood-ratio test (p = .02) was therefore considered unstable and was not interpreted as reliable evidence of selection bias.

**Table S5**

*Assessment of Small-Study Effects Using Egger Regression by Comparator Subgroup (Active Control vs Active Treatment)*

| Outcome | Model | Test Type | *b* | *t* | *p* | 95% CI |
| --- | --- | --- | --- | --- | --- | --- |
| Severity of depression (active control) | End of treatment | Egger Sandwich test | 4.26 | 1.63 | .196 | −3.80, 12.32 |
| Severity of depression (active control) | End of treatment—high RoB studies removed | Standard Egger regression | 2.22 | 1.71 | .230 | −3.37, 7.81 |
| Severity of depression (active control) | All time points | Egger Sandwich test | 4.06 | 1.64 | .202 | −3.92, 12.04 |
| Severity of depression (active control) | All time points—high RoB studies removed | Standard Egger regression | 1.83 | 1.41 | .294 | −3.75, 7.41 |
| Severity of anxiety (active control) | End of treatment | Standard Egger regression | 6.30 | 2.41 | .251 | −26.91, 39.50 |
| Severity of anxiety (active control) | All time points | Standard Egger regression | 0.18 | 0.03 | .983 | −82.82, 83.17 |
| Global distress (active control) | End of treatment | Standard Egger regression | 0.89 | 0.30 | .790 | −11.75, 13.54 |
| Global distress (active control) | All time points | Standard Egger regression | −3.30 | −0.65 | .582 | −25.12, 18.51 |
| Severity of depression (active treatment) | End of treatment | Egger Sandwich test | −1.24 | −1.77 | .203 | −3.92, 1.44 |
| Severity of depression (active treatment) | End of treatment—high RoB studies removed | Egger Sandwich test | −1.47 | −2.07 | .186 | −4.82, 1.87 |
| Severity of depression (active treatment) | All time points | Egger Sandwich test | −0.80 | −0.84 | .478 | −4.38, 2.77 |
| Severity of depression (active treatment) | All time points—high RoB studies removed | Standard Egger regression | −0.85 | −0.81 | .453 | −3.52, 1.83 |
| Severity of anxiety (active treatment) | End of treatment | Standard Egger regression | −2.43 | −2.27 | .151 | −7.04, 2.18 |
| Severity of anxiety (active treatment) | End of treatment—high RoB studies removed | Standard Egger regression | −1.96 | −1.62 | .353 | −17.40, 13.47 |
| Severity of anxiety (active treatment) | All time points | Standard Egger regression | −2.38 | −2.28 | .150 | −6.87, 2.11 |
| Severity of anxiety (active treatment) | All time points—high RoB studies removed | Standard Egger regression | −2.12 | −1.78 | .326 | −17.27, 13.03 |
| Global distress (active treatment) | End of treatment | Egger Sandwich test | −0.76 | −1.20 | .353 | −3.50, 1.98 |
| Global distress (active treatment) | End of treatment—high RoB studies removed | Egger Sandwich test | −0.71 | −1.20 | .381 | −4.03, 2.62 |
| Global distress (active treatment) | All time points | Egger Sandwich test | −0.41 | −0.92 | .448 | −2.23, 1.40 |
| Global distress (active treatment) | All time points—high RoB studies removed | Egger Sandwich test | −0.34 | −0.79 | .525 | −2.57, 1.90 |

*Note.* Where robust variance estimation was used in the primary meta-analysis, the Egger sandwich test was applied; otherwise, the standard Egger regression test was used. The coefficient b represents the slope for study standard error. A statistically significant slope indicates potential funnel-plot asymmetry, which may reflect publication bias. Tests were repeated following removal of studies rated as high risk of bias (high RoB) where indicated. Confidence intervals are reported at the 95% level. Results should be interpreted cautiously in models with a small number of studies, as power to detect asymmetry is limited.

**Table S6**

*Three-Parameter Selection Model (3PSM) Analyses by Comparator Subgroup (Active Control vs Active Treatment)*

| Comparator | Outcome | Timepoint | Bias Adjustment | α | *k* (*p* ≤ α) | δ | *LRT* | *p* |
| --- | --- | --- | --- | --- | --- | --- | --- | --- |
| Versus active control | Severity of depression | All time points |  | 0.025 | 4 | 0.13 | 2.02 | .16 |
| Versus active control | Severity of depression | All time points | High RoB removed | 0.025 | 3 | 0.10 | 2.36 | .12 |
| Versus active control | Severity of depression | All time points |  | 0.05 | 4 | 0.34 | 0.63 | .43 |
| Versus active control | Severity of depression | All time points | High RoB removed | 0.05 | 3 | 0.26 | 0.88 | .35 |
| Versus active control | Severity of depression | End of treatment |  | 0.025 | 3 | 1.87 | 0.21 | .64 |
| Versus active control | Severity of depression | End of treatment | High RoB removed | 0.025 | 2 | 0.74 | 0.02 | .88 |
| Versus active control | Severity of depression | End of treatment |  | 0.05 | 4 | 0.55 | 0.20 | .66 |
| Versus active control | Severity of depression | End of treatment | High RoB removed | 0.05 | 3 | 0.18 | 1.29 | .26 |
| Versus active control | Severity of anxiety | Insufficient clusters to perform 3PSM test. (k = 2) | | | | | | |
| Versus active control | Global distress | All time points |  | 0.025 | 2 | 0.12 | 1.42 | .23 |
| Versus active control | Global distress | All time points |  | 0.05 | 2 | 0.31 | 0.49 | .48 |
| Versus active control | Global distress | End of treatment |  | 0.025 | 2 | 0.43 | 0.26 | .61 |
| Versus active control | Global distress | End of treatment |  | 0.05 | 2 | 1.43 | 0.05 | .82 |
| Versus active treatment | Severity of depression | All time points |  | 0.025 | 1 | 3.51 | 0.81 | .37 |
| Versus active treatment | Severity of depression | All time points | High RoB removed | 0.025 | 1 | 2.71 | 0.47 | .49 |
| Versus active treatment | Severity of depression | All time points |  | 0.05 | 2 | 1.88 | 0.27 | .60 |
| Versus active treatment | Severity of depression | All time points | High RoB removed | 0.05 | 2 | 1.29 | 0.04 | .84 |
| Versus active treatment | Severity of depression | End of treatment |  | 0.025 | 2 | 1.38 | 0.07 | .79 |
| Versus active treatment | Severity of depression | End of treatment | High RoB removed | 0.025 | 2 | 1.03 | 0.00 | .98 |
| Versus active treatment | Severity of depression | End of treatment |  | 0.05 | 3 | 1.06 | 0.00 | .96 |
| Versus active treatment | Severity of depression | End of treatment | High RoB removed | 0.05 | 3 | 0.69 | 0.09 | .76 |
| Versus active treatment | Severity of anxiety | All time points |  | 0.025 | 1 | 0.09 | 1.64 | .20 |
| Versus active treatment | Severity of anxiety | All time points | High RoB removed | 0.025 | 1 | 0.12 | 1.20 | .27 |
| Versus active treatment | Severity of anxiety | All time points |  | 0.05 | 1 | 0.23 | 0.67 | .41 |
| Versus active treatment | Severity of anxiety | All time points | High RoB removed | 0.05 | 1 | 0.34 | 0.32 | .57 |
| Versus active treatment | Severity of anxiety | End of treatment |  | 0.025 | 1 | 0.22 | 0.64 | .42 |
| Versus active treatment | Severity of anxiety | End of treatment | High RoB removed | 0.025 | 1 | 0.39 | 0.22 | .64 |
| Versus active treatment | Severity of anxiety | End of treatment |  | 0.05 | 1 | 0.61 | 0.04 | .84 |
| Versus active treatment | Severity of anxiety | End of treatment | High RoB removed | 0.05 | 1 | 1.19 | 0.01 | .93 |
| Versus active treatment | Global distress | All time points |  | 0.025 | 2 | 1.08 | 0.00 | .95 |
| Versus active treatment | Global distress | All time points | High RoB removed | 0.025 | 2 | 0.88 | 0.01 | .93 |
| Versus active treatment | Global distress | All time points |  | 0.05 | 3 | 0.60 | 0.17 | .68 |
| Versus active treatment | Global distress | All time points | High RoB removed | 0.05 | 3 | 0.37 | 0.54 | .46 |
| Versus active treatment | Global distress | End of treatment |  | 0.025 | 2 | 1.05 | 0.00 | .97 |
| Versus active treatment | Global distress | End of treatment | High RoB removed | 0.025 | 2 | 0.85 | 0.01 | .91 |
| Versus active treatment | Global distress | End of treatment |  | 0.05 | 2 | 2.96 | 0.70 | .40 |
| Versus active treatment | Global distress | End of treatment | High RoB removed | 0.05 | 2 | 2.33 | 0.37 | .54 |

*Note.* Results from three-parameter selection models (3PSM) using step-function weight functions with cut-points at α = .025 and α = .05. *k* (p ≤ α) denotes the number of effects within the lower significance interval. δ represents the estimated relative weight (selection parameter) for effects in the higher *p*-value interval relative to the lowest interval. *LRT* denotes the likelihood ratio test comparing the selection model with the no-selection model. “High RoB removed” indicates models re-estimated after excluding studies rated at high risk of bias.
